# Determining the Proximity Effect-Induced Magnetic Moment in Graphene by Polarized Neutron Reflectivity and X-ray Magnetic Circular Dichroism

**DOI:** 10.1021/acsami.2c02840

**Published:** 2023-04-24

**Authors:** Razan O. M. Aboljadayel, Christy J. Kinane, Carlos A. F. Vaz, David M. Love, Robert S. Weatherup, Philipp Braeuninger-Weimer, Marie-Blandine Martin, Adrian Ionescu, Andrew J. Caruana, Timothy R. Charlton, Justin Llandro, Pedro M. S. Monteiro, Crispin H. W. Barnes, Stephan Hofmann, Sean Langridge

**Affiliations:** † Cavendish Laboratory, Physics Department, University of Cambridge, Cambridge CB3 0HE, United Kingdom; ‡ ISIS Facility, STFC Rutherford Appleton Laboratory, Harwell Science and Innovation Campus, Oxon OX11 0QX, United Kingdom; § Swiss Light Source, Paul Scherrer Institut, Villigen PSI 5232, Switzerland; ∥ Department of Engineering, University of Cambridge, Cambridge CB3 0FA, United Kingdom

**Keywords:** graphene, XMCD, PNR, magnetism, heterostructure

## Abstract

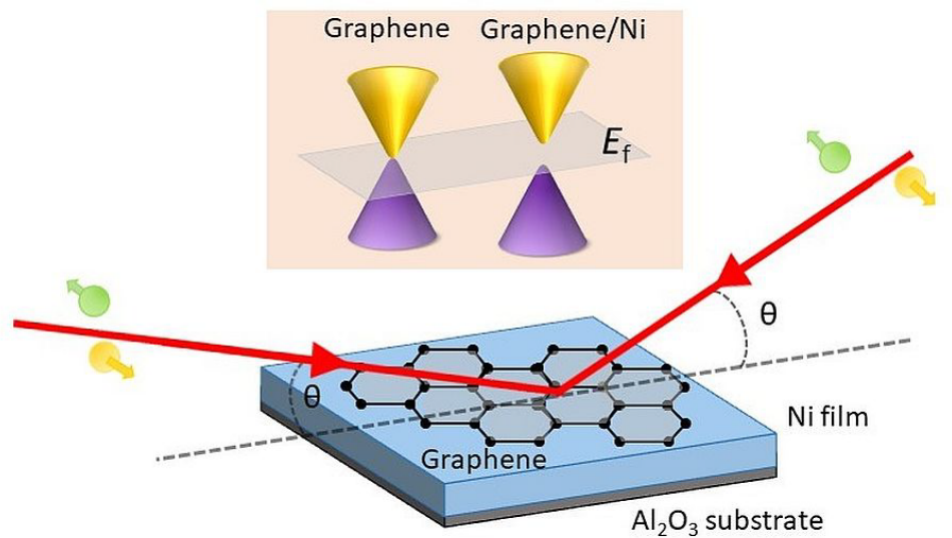

We report the magnitude of the induced magnetic moment in CVD-grown epitaxial and rotated-domain graphene in proximity with a ferromagnetic Ni film, using polarized neutron reflectivity (PNR) and X-ray magnetic circular dichroism (XMCD). The XMCD spectra at the C *K*-edge confirm the presence of a magnetic signal in the graphene layer, and the sum rules give a magnetic moment of up to ∼0.47 μ_B_/C atom induced in the graphene layer. For a more precise estimation, we conducted PNR measurements. The PNR results indicate an induced magnetic moment of ∼0.41 μ_B_/C atom at 10 K for epitaxial and rotated-domain graphene. Additional PNR measurements on graphene grown on a nonmagnetic Ni_9_Mo_1_ substrate, where no magnetic moment in graphene is measured, suggest that the origin of the induced magnetic moment is due to the opening of the graphene’s Dirac cone as a result of the strong C p_*z*_-Ni 3d hybridization.

## Introduction

Graphene is a promising material for many technological and future spintronic device applications such as spin-filters,^1−7^ spin-valves, and spin field-effect transistors due to its excellent transport properties.^8,9^ Graphene can have an intrinsic charge carrier mobility of more than 200 000 cm^2^ V^–1^ s^–1^ at room temperature (RT)^10^ and a large spin relaxation time as a result of its long electron mean free path and its negligible spin–orbit and hyperfine couplings.^2,11^

Manipulating spins directly in the graphene layer has attracted great attention as it opens new ways for using this 2D material in spintronics applications.^2,12,13^ This has been realized via various approaches such as through the proximity-induced effect,^2,14−17^ chemical doping of the graphene surface,^11^ or through a chemically induced sublattice.^18^ Here, we report the feasibility of the first method in utilizing the exchange coupling of local moments between graphene and a ferromagnetic (FM) material to induce a magnetic moment in graphene.

Graphene is a zero-gap semiconductor because the π and π* bands meet at the Fermi energy (*E*_F_), at the corner of the graphene’s Brillouin zone (*K* points), that is, at degenerate points forming the Dirac point (*E*_D_), where the electronic structure of these bands can be described using the tight-binding model.^19,20^ However, the adsorption of graphene on a strongly interacting metal distorts its intrinsic band structure around *E*_D_. This is a result of the overlap of the graphene’s valence band with that of the metal substrate due to the breaking of degeneracy around *E*_D_ in a partially filled d-metal, as discussed in the universal model proposed by Voloshina and Dedkov.^21^ Their model was supported by density functional theory calculations and proven experimentally using angle-resolved photoemission spectroscopy.^19,21−26^ Furthermore, a small magnetic signal was detected in the X-ray magnetic circular dichroism (XMCD) spectra of the graphene layer in proximity with a FM transition metal (TM) film, suggesting that a magnetic moment is induced in the graphene.^2,14,27,28^ However, no direct quantitative analysis of the total induced magnetic moment has been reported.

It is widely accepted that graphene’s C atoms are assembled on top of close-packed (111) surfaces in what is known as the top-fcc configuration, where the C atoms are placed on top of the atoms of the first and third layers of the TM substrate.^21,23,24^ The strength of the graphene–TM interaction is influenced by the lattice mismatch, the graphene–TM bond length, and the position of the d orbital of the TM relative to *E*_F_. Therefore, a Ni(111) substrate was used as the FM because it has a small lattice mismatch of −1.2%, a bond length of 2.03 Å, and their d orbitals are positioned ∼1.1 eV below *E*_F_ (i.e., forming π–d hybrid states around the *K* points).^21−23,29^ Epitaxial and rotated-domain graphene structures were investigated because rotated graphene is expected to interact more weakly with the TM film underneath. This is a result of the loss of epitaxial relationship and a lower charge transfer from the TM due to the missing direct Ni_top_–C interaction and the smaller region covered by an extended graphene layer as a result of the 3° rotation between the graphene and Ni.^23,30−32^ Therefore, a smaller magnetic moment is expected to be induced in rotated-domain graphene.

We have studied the structural, magnetic, and electronic properties of epitaxial- and rotated-domain graphene grown on Ni films, confirmed the presence of a magnetic moment in graphene by element-specific XMCD, and measured the induced magnetic moment at 10 and 300 K for rotated and epitaxial graphene using polarized neutron reflectivity (PNR).

To our knowledge, our attempt is the first reported approach in using PNR and XMCD sum rules combined to estimate the total induced magnetic moment in graphene. This is due to the thinness of graphene, which is close to the resolution of the PNR technique, and the difficulty in processing the XMCD C *K*-edge signal due to the contribution of the carbon contamination in the beamline optics. We attribute the presence of an induced magnetic moment in graphene to the hybridization of the C p_*z*_ orbital with the 3d bands of the TM, which is supported by additional PNR measurements on graphene grown on a nonmagnetic Ni_9_Mo_1_(111) substrate, where no magnetic moment is detected in the graphene layer.

## Results and Discussion

### Raman Spectroscopy Measurements

To evaluate the quality, number of graphene layers, doping, and defect density in the grown graphene samples, we used Raman spectroscopy, which is a nondestructive technique known to be particularly sensitive to the structural and electronic properties of graphene.^33,34^ For these measurements, the graphene layer was first transferred by a chemical etching process from the metallic film onto a Si substrate with a thermally oxidized SiO_2_ layer similar to that reported in ref (35). This was done to avoid loss in the resonance conditions due to the strong chemical interaction between the graphene π orbital and the d-states of Ni and Ni_9_Mo_1_, which also alters the graphene’s p_*z*_ orbitals (see the Experimental Section for further details).

**Figure 1:**
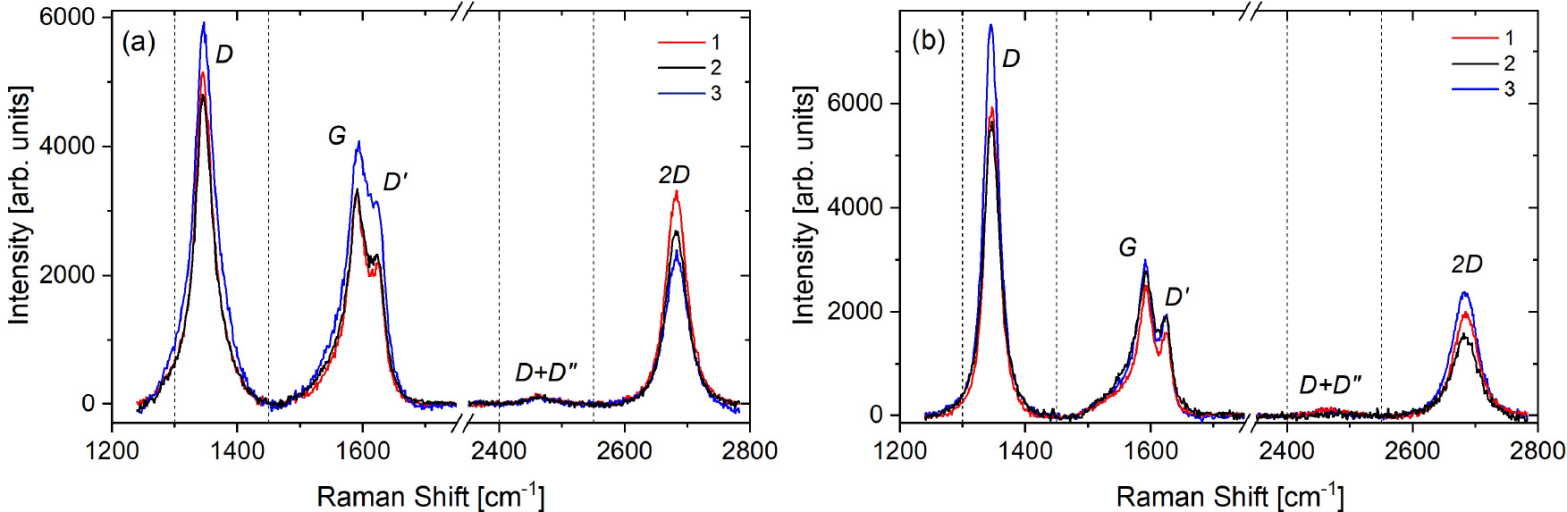
Room-temperature Raman spectroscopy measurements taken at three different regions (1–3) after transferring the graphene from the Ni film to a Si/SiO_2_ wafer for (a) epitaxial and (b) rotated graphene, showing the graphene’s characteristic peaks. The dashed vertical lines separate the regions of the different peaks.

Figure 1 shows the Raman scans taken at three different regions of the graphene after being transferred from the Ni films (see the Experimental Section). All of the spectra possess the D, G, D′, D + D″, and 2D peaks.^36,37^ Although all of the 2D peaks shown in Figure 1 were fitted with single Lorentzians, they have a relatively broad full-width at half-maximum (FWHM). The average FWHM values of the 2D peak of epitaxial and rotated-domain graphene transferred from the Ni film are 40.8 and 46.2 cm^–1^, respectively. Furthermore, the spectra of both samples show a high *I*_2D_/*I*_G_ ratio (average of 1.49 for epitaxial graphene and 2.35 for rotated graphene). The variation in the spectra of each sample, the presence of second-order and defect-induced peaks, the large FWHM of the 2D peak, and the high *I*_2D_/*I*_*G*_ ratio could be a result of the chemical etching and transfer process (see the Sample Preparation) or the chemical doping from the HNO_3_ used to etch the metallic films. Therefore, it is difficult to estimate the number of graphene layers on the basis of the position of the G and 2D peaks and *I*_2D_/*I*_G_ ratio. However, the SEM scan and LEED diffraction pattern show a single epitaxial graphene layer grown on Ni(111) (see the Experimental Section). The broader FWHM of the 2D peak and the higher average *I*_2D_/*I*_G_ ratio in the rotated graphene as compared to the epitaxial structure could be attributed to the formation of more defective or turbostratic graphene (multilayer graphene with relative rotation between the layers) as a result of the occasional overlap of the graphene domains.^38^ The Raman spectra for the graphene/Ni_9_Mo_1_ sample, as well as the full list of the peak positions and the 2D average FWHM of all of the measured samples, are provided in the Supporting Information.

### X-ray Magnetic Circular Dichroism (XMCD)

**Figure 2:**
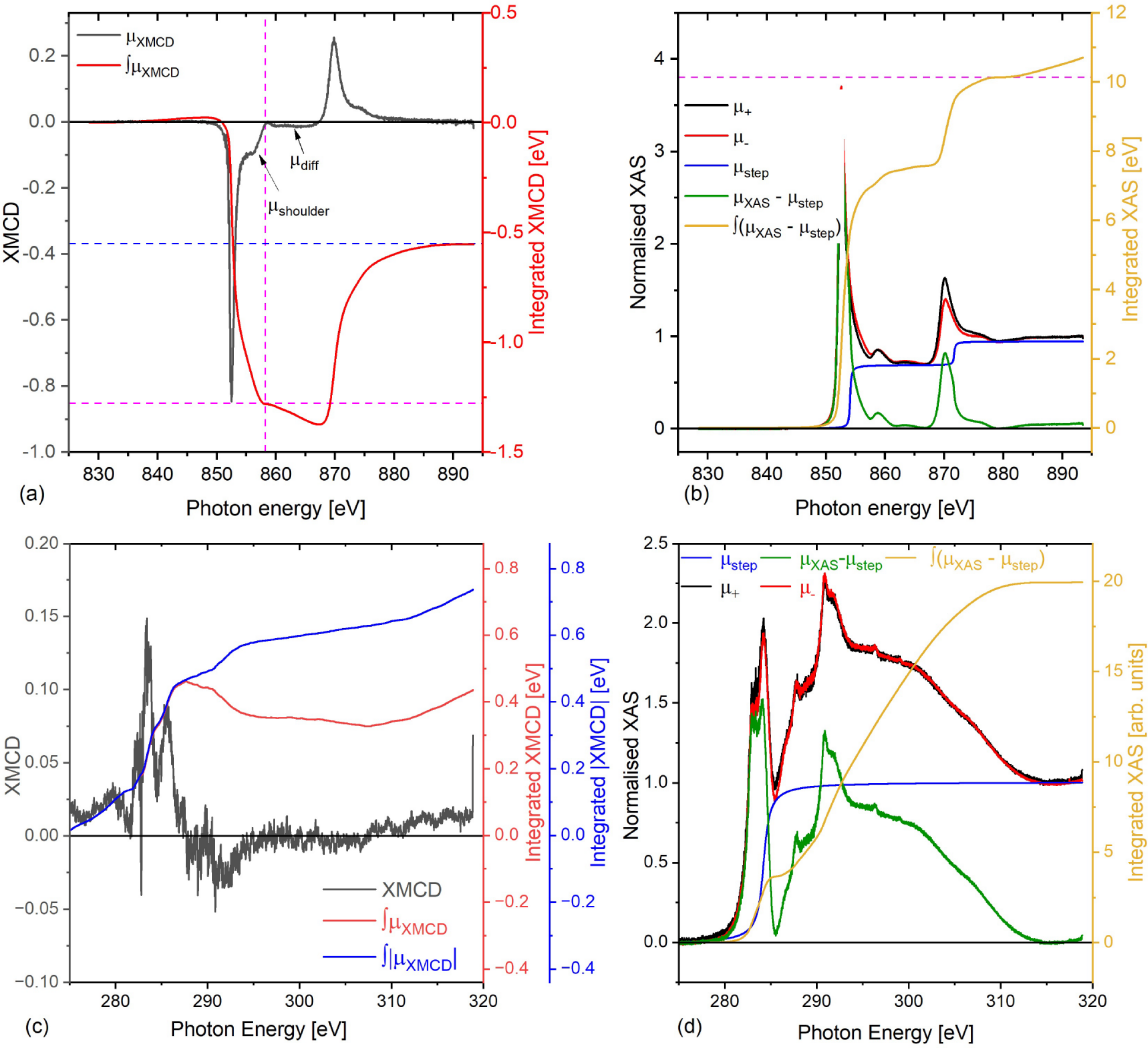
X-ray absorption spectra for circular polarized light for the rotated graphene/Ni sample measured at 300 K: (a,b) XMCD and XAS spectra for the Ni *L*_2,3_-edge. (c,d) XMCD and XAS spectra for the graphene layer. The vertical and horizontal dashed lines in (a) indicate the maximum integration range over the *L*_2,3_ peak and the values used for the calculation of *m*_o_ and *m*_s_, respectively.

The X-ray absorption spectra (μ_XAS_) and the XMCD response (μ_XMCD_) at the Ni *L*_2,3_-edge in the rotated graphene/Ni sample are shown in Figure 2. The spectra show no sign of oxidation, proving that graphene acts as a good passivation layer against oxidation.^39,40^ The region between the *L*_3_ and *L*_2_ edges with a constant negative intensity is known as the diffuse magnetism region, μ_diff_, and it has been observed and reported for the Co, Ni, and Fe XMCD spectra.^41,42^ μ_diff_ is expected to arise as a result of the opposite spin directions for the 4s and 3d electrons, interstitial and sp-projected magnetic moments, and the fact that it couples antiferromagnetically to the sample’s total magnetic moment in 3d elements.^41^ Although μ_diff_ has been reported to contribute to about −7% to the total magnetic moment in Ni,^41,43^ because the sum rule does not account for μ_diff_, the integration range over the *L*_3_ was stopped just before μ_diff_ for the calculation of the orbital magnetic moment, *m*_o_, and the spin magnetic moment, *m*_s_ (858.7 eV). However, the main *L*_3_ peak and the shoulder, μ_shoulder_, are due to multiple initial-state configurations, 3d^8^ and 3d^9^, respectively, and therefore they were accounted for in the sum rule calculations.^41^

Although the nonresonant contribution was subtracted from the μ_+_ and μ_–_ spectra, a higher background is measured at the post-edge (*E* > 880 eV). This tail has been excluded from the sum rules as it is considered part of the nonresonant contribution. The calculated *m*_o_ and *m*_s_ values are 0.084 and 0.625 μ_B_/Ni atom, respectively, and thus *m*_total_ is 0.709 μ_B_/Ni atom (see the Supporting Information for the expressions for *m*_o_, *m*_s_, and *m*_total_ at the *L*-edge). Considering the 20% accuracy of the XMCD technique in estimating the magnetic moments of materials, the results obtained for Ni are consistent with the values reported in the literature.^44,45^

The C *K*-edge spectra for the rotated graphene/Ni sample are shown in Figure 2c and d. For the C *K*-edge, higher sources of errors are expected in the XMCD estimation attributed to the difficulty of applying the sum rules to the C *K*-edge spectra in comparison with that for the Ni *L*_2,3_-edge. For instance, various studies have been reported for Ni,^14,24,43,44,46−48^ which can be used as references for our measurements, but the application of the sum rules has not been reported for graphene before. Also, the number of holes, *n*_h_, has not been measured for C previously. Moreover, the gyromagnetic factor (*g*) of the graphene was found to be different depending on the underlying substrate,^49−51^ and it has not been reported for graphene on Ni. It is also noteworthy to mention the difficulty associated with measuring the C *K*-edge due to the C contamination of the optical elements, which appear as a significant reduction in the incoming intensity at this particular energy.

Nonetheless, we can obtain an upper limit to the orbital moment of the graphene layer by integrating the modulus of the dichroic signal, |μ_XMCD_|, which is shown in Figure 2c, blue curve. Although the magnetic dichroism response is expected mainly at the peak corresponding to the 1s → π* transition as a result of the C p_*z*_–Ni 3d hybridization,^14^ a small magnetic signal is observed at the 1s → σ* transition peak as well; a similar behavior was reported for graphene/2 ML Co/Ir(111).^25^ The calculated upper bound *m*_o_ for graphene is 0.062 μ_B_/C atom using *n*_h_ = 4, which corresponds to an *m*_s_ of 0.412 μ_B_/C atom, using *g* = 2.3, which is the value reported for graphene grown on SiC.^49^ Therefore, the upper limit of *m*_total_ of the rotated-domain graphene grown on Ni is ∼0.474 μ_B_/C atom (see the Supporting Information for the expressions of *m*_o_, *m*_s_, and *m*_total_ at the *K*-edge).

Despite the large uncertainties expected for the estimated graphene moments, the XMCD results demonstrate the presence of magnetic polarization in graphene.

Next, we turn to PNR to combine all of the information from complementary techniques, XMCD, Raman, and SEM, to obtain a more precise value for the magnetic moment in the graphene.

### Polarized Neutron Reflectivity

PNR experiments were carried out to measure the magnetic properties of each layer of the samples individually and to determine the value of the induced magnetic moment in graphene quantitatively.

**Figure 3:**
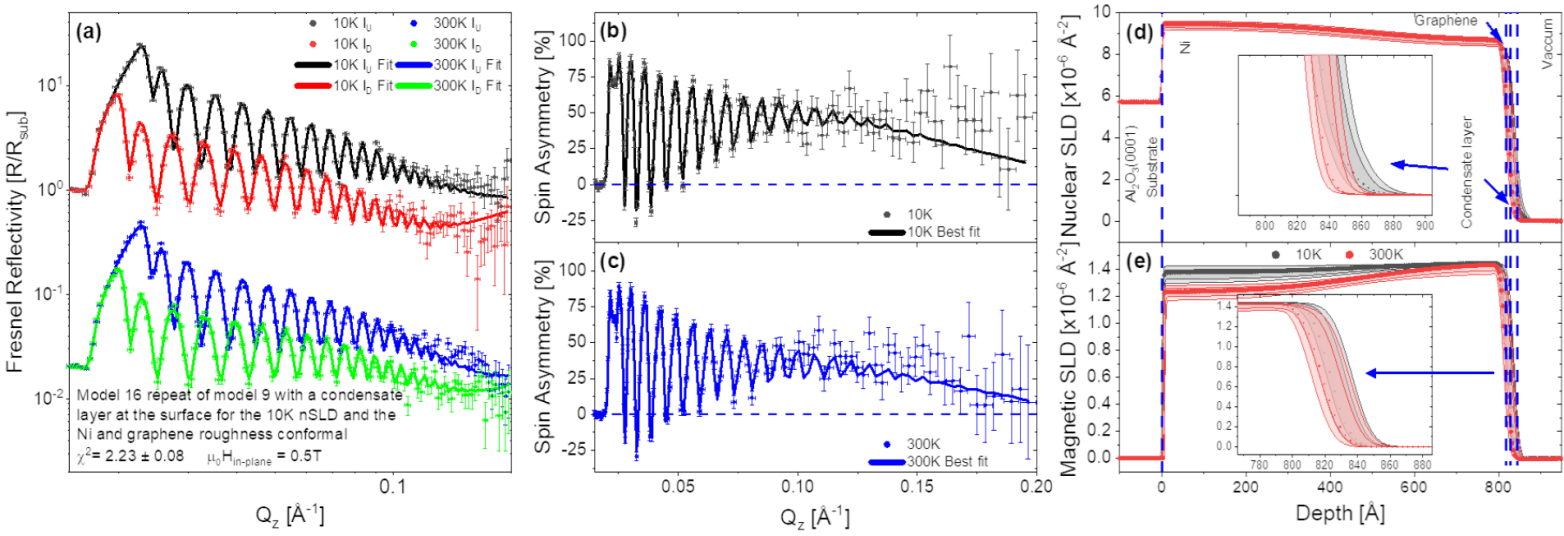
PNR (model 16) results for rotated-domain graphene. A liquid air “condensate” layer has been added to the surface of the 10 K model only, not included in the 300 K model. The top Ni layer and graphene roughnesses have been linked to make them conformal. (a) Fresnel reflectivity at 10 and 300 K, (b,c) spin asymmetries, (d) the nuclear scattering length density (nSLD) profiles at 10 and 300 K, and (e) the magnetic scattering length density (mSLD) profiles. The gray banded regions around the SLD lines are the 95% Bayesian confidence intervals. The room temperature Fresnel reflectivity has been shifted by a factor of 50 for ease of display.

**Figure 4:**
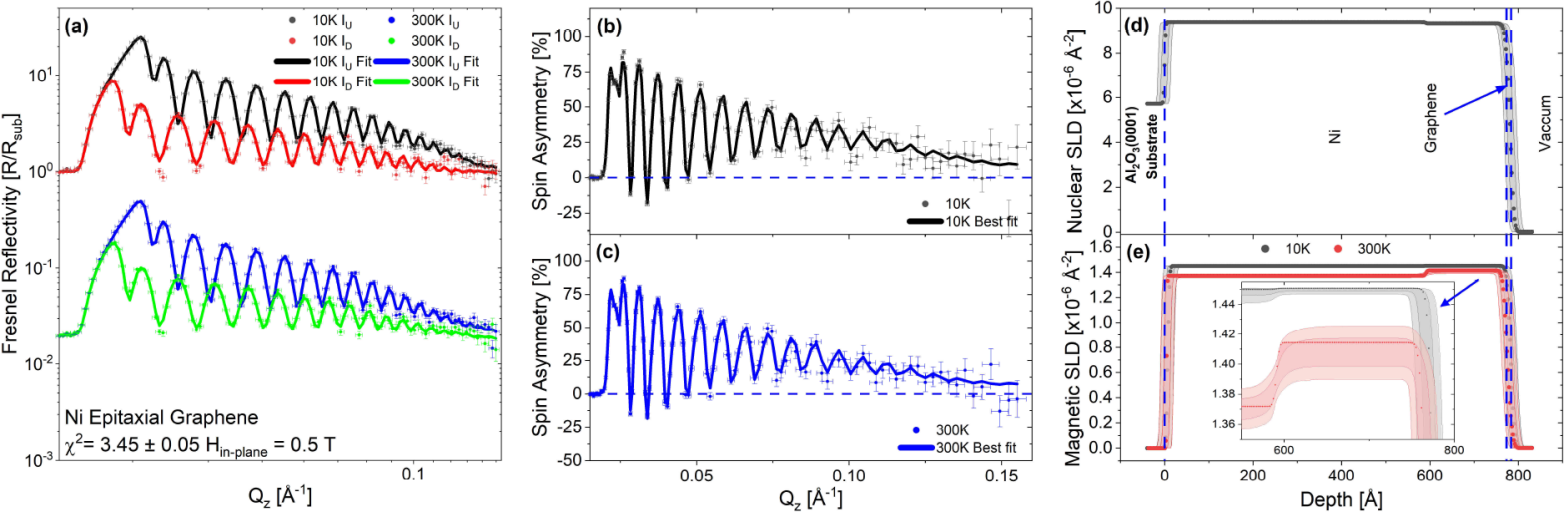
Ni layer split into two regions for the epitaxial graphene/Ni sample. The top Ni layer and graphene roughnesses have been linked to make them conformal. (a) Fresnel reflectivity for 10 and 300 K, (b,c) spin asymmetries, (d) the nuclear scattering length density (nSLD) profile, and (e) the magnetic scattering length density (mSLD) profile. The room temperature Fresnel reflectivity has been shifted by a factor of 50 for ease of display.

The PNR results for the rotated-domain graphene/Ni(111) and epitaxial graphene/Ni(111) samples, measured at 10 and 300 K, are displayed in Figures 3 and 4, respectively. Panel (a) for each figure shows the Fresnel reflectivity profiles, and panels (b) and (c) the spin asymmetry (*SA* = [*R*_+_ – *R*_–_]/[*R*_+_ + *R*_–_], where *R*_+_ and *R*_–_ are the spin-up and spin-down neutron specular reflectivities, respectively). *SA* scales with the magnetic signal. A flat *SA* line at zero, shown as a blue dashed line, represents no net magnetic induction present in the system. Panel (d) displays the nuclear scattering length density (nSLD) for the sample structure. This structure is shared at both temperatures in the co-refinement, and panel (e) shows the magnetic scattering length density (mSLD) for each temperature.

The fitting procedure is fully described in the Supporting Information, which contains the various models used to fit the rotated graphene/Ni sample. The 10 and 300 K data shown in Figures 3 and 4 were fitted simultaneously with a shared nSLD and independent mSLD. The bulk of the fitting model selection was done on the rotated graphene/Ni sample. The study presented in the Supporting Information shows the importance of prior knowledge of the sample properties to obtain the best PNR fit to estimate the induced magnetic moment in graphene. We used the information obtained from the structural characterizations (SEM and Raman spectroscopy) to obtain a lower bound on the graphene layer thickness. The fit tends to shrink the graphene thickness to less than one monolayer, if unrestrained, which does not agree with the SEM and Raman scans. This could be due to the limited *Q* (wavevector transfer) range measured in the time available for the experiment, as the fit is found to rely strongly on the high *Q* statistics. It should be noted that in model 16, shown in Figure 3, the magnetic moment of the graphene layer was allowed to fit below zero, but the analysis always required a nonzero value for the magnetic moment to obtain a good fit with low uncertainty, which is consistent with the XMCD results. The unrestrained graphene thickness models are shown in the Supporting Information. It is noteworthy that the improvement in the figure of merit (χ^2^) for the unconstrained to the constrained graphene thickness is within the error bar of each other. However, the results show that the graphene thickness has only a subtle influence, if any, on the amount of the induced magnetization as can be deduced from the values of the measured magnetic moments. To get greater certainty on the model selection given the data, we used a nested sampler^52,53^ to calculate the Bayesian evidence taking into account the structural information, providing a high degree of confidence in the final fit model as shown in the Supporting Information.

Additional scenarios were also tested. For example, oxidation of the Ni layer and the formation of Ni-carbide were examined by embedding an intermediate NiO and Ni_2_C layer, respectively, at the interface between the Ni and graphene, but this led to poorer fits, and the best results, shown in Figures 3 and 4, were achieved using a simpler model: substrate/FM layer split into two regions/graphene (see the modeling methodology in the Supporting Information).

The results of the fits to the PNR data are summarized in Table 1. Interestingly, both the rotated and the epitaxial grown graphene systems have very similar moments in the graphene at both 10 and 300 K, counter to the initial hypothesis that the coupling between the graphene and Ni(111) would be weaker in the rotated-domain case. The 95% confidence intervals, as shown in the parentheses of Table 1 for both the epitaxial and the rotated-domain graphene moments, are quite large and overlap, indicating that we do not have the sensitivity to confirm or dismiss if this hypothesis holds. Both Ni(111) samples have the full Ni moment at 10 K, which is only slightly reduced at 300 K. This reduction is associated with a nuclear gradient across the Ni(111) film. In the rotated-domain sample, this gradient, as shown in Figure 3d, starts with a SLD value close to the bulk nSLD of Ni (9.414 × 10^–6^ Å^–2^)^54^ and reduces in size toward the surface, which is required to fit the lower *Q* features and allow the higher *Q* features to converge, paramount to getting certainty on the thin graphene layers. Consequently, the mSLD also has a gradient that oppositely mirrors the nSLD at 300 K and reaches the full moment for Ni (0.6 μ_B_/Ni atom equivalent to an mSLD = 1.4514 × 10^–6^ Å^–2^) near the surface and being slightly reduced near the substrate. At 10 K the magnetic gradient vastly reduces, with the Ni layer becoming almost uniformly magnetic; we speculate that this may be a result of the lower temperature overcoming any change in the Curie temperature due to strain effects across the film. Similar effects have been observed in other systems due to doping and strain both versus temperature.^55−57^ The Ni(111) used for the epitaxial graphene displays a much weaker structural gradient, being almost uniform across the Ni thickness, but has the same general trends. We attribute the origin of the difference in the nSLD profiles to the fact that the samples were deposited at different times and have different strains, following the growth recipe described in the Sample Preparation. Again, there is a slight difference in the mSLD curves from 300 to 10 K in the epitaxial graphene sample.

**Table 1:** Summary of the PNR Results for the Rotated Graphene/Ni, Epitaxial Graphene/Ni, and Graphene/Ni_9_Mo_1_ Samples Using Model 16: Sapphire/FM Layer Split into Two Regions/Graphene^a^

	FM layer1 + FM layer2			graphene	
sample	temperature (K)	thickness (nm)	magnetic moment (μ_B_/atom)	thickness (nm)	magnetic moment (μ_B_/atom)
rotated Gr/Ni	10	82.8 (80.8, 82.8)	0.61 (0.61, 0.62)	0.82 (0.81, 1.2)	0.41 (0.28, 0.48)
	300		0.58 (0.57, 0.58)		0.23 (0.02, 0.41)
epitaxial Gr/Ni	10	77.4 (77.0, 77.6)	0.60 (0.60, 0.61)	0.99 (0.82, 1.2)	0.41 (0.25, 0.51)
	300		0.580 (0.576, 0.583)		0.15 (0.05, 0.46)
Gr/Ni_9_Mo_1_	10	77.1 (76.4, 77.7)	0.060 (0.056, 0.066)	1.0 (0.81, 1.2)	0.34 (0.06, 0.56)
	300		0.001 (−0.003, 0.006)		–0.01 (−0.04, 0.26)

a The top Ni layer and graphene roughnesses have been linked to make them conformal. The values in the parentheses are the lower and upper 95% Bayesian confidence limits.^62^

The Ni_9_Mo_1_ sample was used to clarify whether the induced magnetic moments in graphene are due to the C p_*z*_-Ni 3d hybridization, which opens the Dirac cone as a result of the degeneracy breaking, as postulated in refs (2), (19), (21), (24), and (29), or because of electron transfer (spin doping) and surface reconstruction, which distorts the d band of the TM as for fullerene/nonmagnetic TM, as proposed in refs (58−60). For this purpose, a Ni_9_Mo_1_ film was used with the aim of preserving the fcc crystal structure of Ni while suppressing its magnetization, as suggested in ref (61). Because the Ni is doped by 10% only, the lattice mismatch and bond length to graphene are expected to be similar to those for graphene/Ni(111) sample, but the d-orbital position is considerably downshifted with respect to *E*_F_. The growth procedure and the structural properties of the sample are discussed in detail in the Experimental Section.

**Figure 5:**
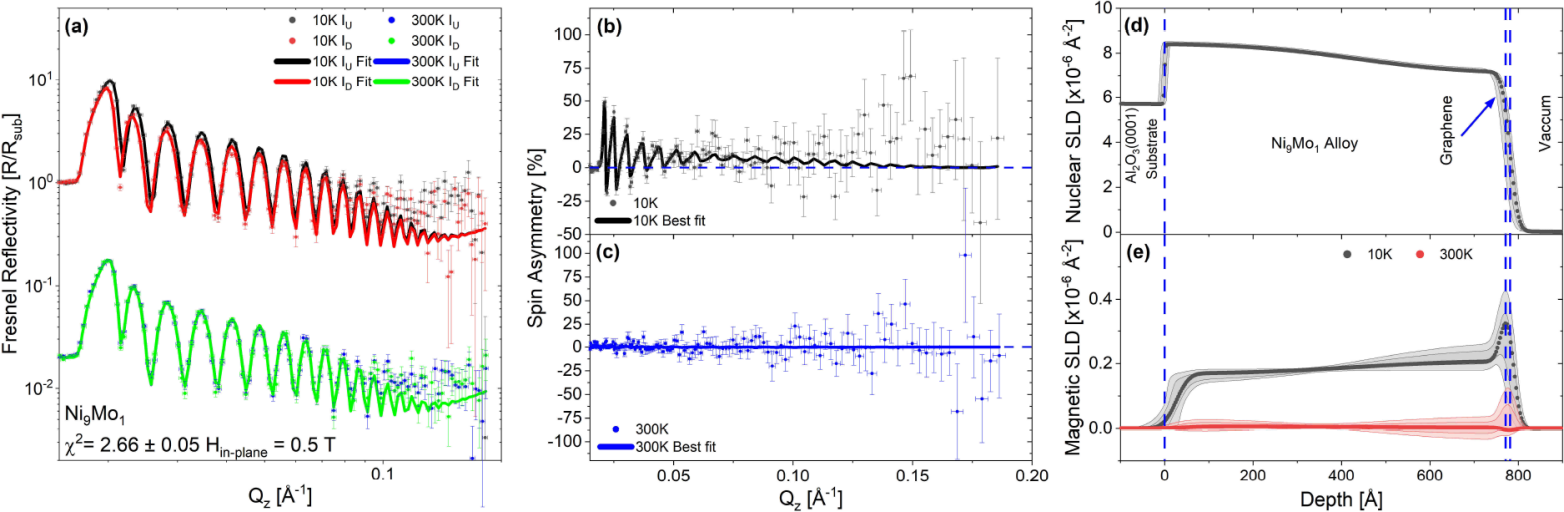
Ni_9_Mo_1_ split into two regions for the graphene/Ni_9_Mo_1_ sample. The top Ni_9_Mo_1_ layer and graphene roughnesses have been linked to make them conformal. (a) Fresnel reflectivity for 10 and 300 K, (b,c) spin asymmetries, (d) the nuclear scattering length density (nSLD) profile, and (e) the magnetic scattering length density (mSLD) profile. The room temperature Fresnel reflectivity has been shifted by a factor of 50 for ease of display.

The results of the PNR measurements of the graphene/Ni_9_Mo_1_ sample are shown in Figure 5. Again, there is an atomic density gradient across the Ni_9_Mo_1_ film. At 10 K, a small but detectable spin splitting and a minute variation in the *SA* are observed. Surprisingly, a higher magnetization is detected in graphene (0.34 (0.06, 0.56) μ_B_/C atom) than in the Ni_9_Mo_1_ film (0.060 (0.056, 0.066) μ_B_/C atom), but the large values of the 95% confidence, reflecting the limited *Q* range and low counting statistics in the data, suggest that this effect may not be real. This is visible in the mSLD curve in Figure 5e as a spike in the magnetism at the surface with a very large error bar. All we can ascertain is that there is a small moment in the Ni_9_Mo_1_ and a nonzero moment in the graphene. The origin of the small residual moment at 10 K could arise from clusters of unalloyed Ni throughout the layer that become ferromagnetic at low temperature, which then polarize the graphene as per the Ni(111) samples. At 300 K, both the alloy and the graphene have effectively zero moment within the 95% confidence intervals. Therefore, the results support the hypothesis of the universal model, whereby the measured induced magnetic moment in graphene is due to the opening of the *E*_D_ rather than the distortion of the d band. This is because no magnetic moment is detected in the Ni_9_Mo_1_ nor in the graphene layer. If the induced magnetic moment in graphene was due to the electron transfer and surface reconstruction, we would have detected magnetism in graphene grown on Ni_9_Mo_1_.

The PNR fits have shown that at 10 K a magnetic moment of ∼0.41 μ_B_/C atom was induced in both the rotated-domain and the epitaxial graphene grown on Ni films. These results indicate larger moments than previously reported.^14,27^ In ref (27), Mertins et al. estimated the magnetic moment of graphene to be 0.14 ± 0.3 μ_B_/C atom when grown on a hcp Co(0001) film. They obtained this value by comparing the XMCD signal of graphene to that of the underlayer Co film.^27^ Weser et al. suggested a similar value (0.05–0.1 μ_B_/C atom) for the magnetic moment induced in a monolayer of graphene grown on a Ni(111) film.^14^ However, their assumption was based on comparing the graphene/Ni system with other C/3d TM structures, such as a C/Fe multilayer with 0.55 nm of C,^63^ and carbon nanotubes (CNTs) on a Co film,^64^ where magnetic moments of 0.05 and 0.1 μ_B_/C atom were estimated, respectively. However, it is difficult to compare graphene-based heterostructures with other C allotropes/TM systems. This is because the Dirac cone is a characteristic feature of graphene and CNTs of the carbon allotropes. Therefore, one cannot exclude that a different mechanism other than the break of degeneracy around *E*_D_ may be responsible for the magnetic moment detected in the C layer of a C/Fe multilayer system. However, although Dirac cones exist in CNTs, for the CNTs/Co heterostructure a direct quantitative analysis of the induced magnetic moment was not possible from the MFM images reported in ref (64). In contrast, our combined technique approach using PNR along with XMCD, Raman, and SEM provides a more precise estimation of the induced magnetic moment in graphene.

## Conclusion

In summary, we have successfully grown graphene by chemical vapor deposition (CVD) on different TM substrates. Induced magnetic moment in rotated-domain graphene as a result of the proximity effect in the vicinity of a FM substrate was detected by element-specific XMCD measurements at the C *K*-edge. PNR experiments were carried out to determine the magnitude of the magnetic moment detected by XMCD. Although a higher magnetic moment was expected to be induced in the epitaxial graphene/Ni sample, the PNR results indicate that the epitaxial graphene film had a magnetic moment of ∼0.41 μ_B_/C atom, similar to that of rotated-domain graphene. Both values are higher than those predicted in other studies.^14,63,64^ PNR measurements on graphene/Ni_9_Mo_1_ support the universal model proposed by Voloshina and Dedkov, where the induced magnetic moment in graphene arises as a result of the opening of the graphene’s Dirac cone as a result of the strong C p_*z*_-Ni 3d hybridization. The PNR results, combined with the other complementary techniques presented here, provides the first quantitative estimation of the induced magnetization in graphene.

## Experimental Section

### Sample Preparation

The sample preparation procedure involved two stages: the growth of the TM films using magnetron sputtering and the growth of graphene by CVD.

**Figure 6:**
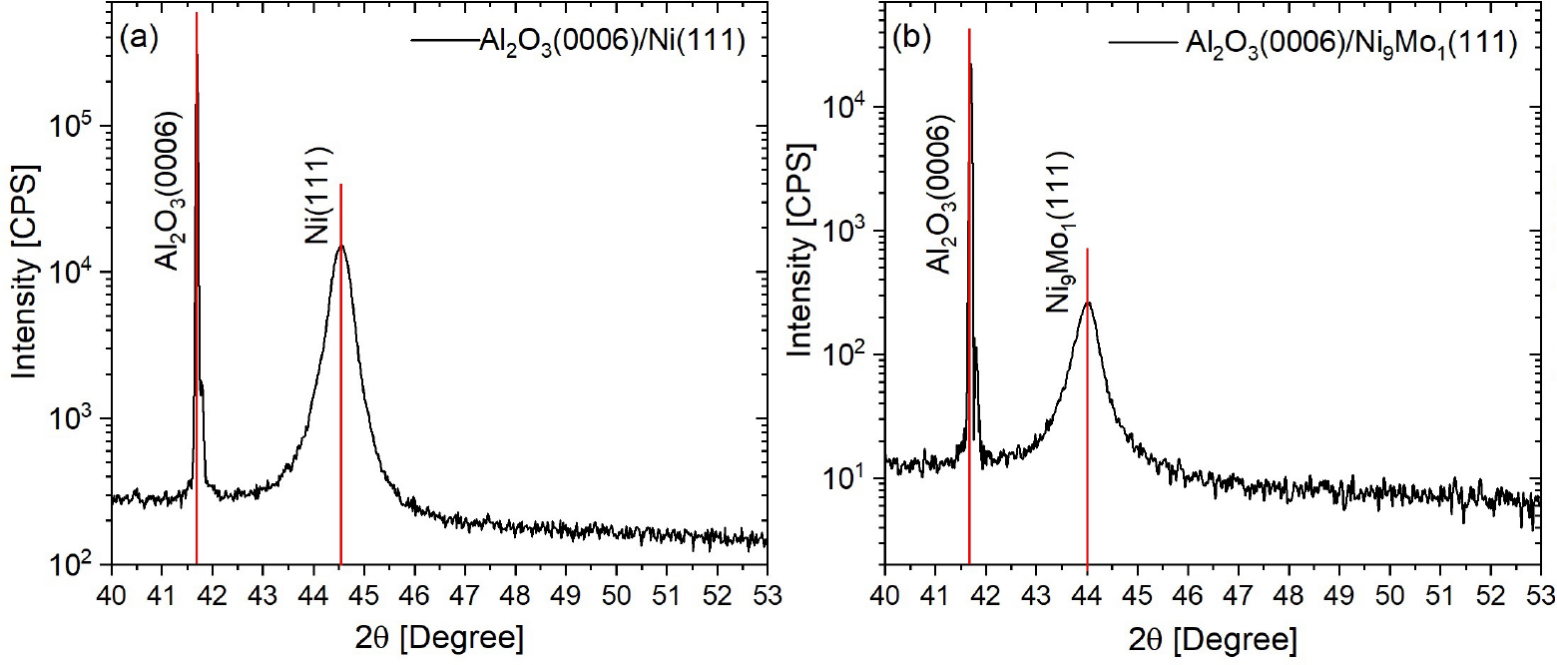
X-ray diffraction measurements (scanning range of 40–53°) of highly textured films: (a) Al_2_O_3_(0001)/Ni(111) and (b) Al_2_O_3_(0001)/Ni_9_Mo_1_(111).

The TM films were deposited at RT on 1 mm thick Al_2_O_3_(0001) substrates using a CEVP magnetron sputtering chamber with a base pressure of (1.2–2) × 10^–8^ mTorr. The thick substrates were used to reduce the possibility of sample deformation, which could affect the reflectivity measurements. The deposition of the TM films was performed using 99.9% pure Ni and Ni_9_Mo_1_ targets. A DC current of 0.1 A and a constant flow of pure argon of 14 sccm were used to grow 80 nm of highly textured Ni(111) and Ni_9_Mo_1_(111) films at a rate of 0.02 nm s^–1^ in a plasma pressure of 2 mTorr (3 mTorr for Ni_9_Mo_1_). Figure 6 shows the X-ray diffraction (XRD) measurements of the deposited films acquired with a Bruker D8 Discover HRXRD with a Cu Kα monochromatic beam (40 kV, 40 mA). The scans show highly textured pure films oriented in the [111] direction for Ni and Ni_9_Mo_1_ films.

The samples were then transferred into a CVD system for the growth of graphene directly on Ni(111) and Ni_9_Mo_1_(111) films on ∼2 cm × 2 cm substrates. Growth recipes similar to those reported by Patera et al.^30^ were adapted to obtain epitaxial and rotated-domain graphene directly on the Ni film. For the Ni_9_Mo_1_ film, rotated-domain graphene was grown by first introducing pure H_2_ gas at a rate of 200 sccm to the CVD chamber with a base pressure of 2.7 × 10^–6^ mbar. The CVD growth chamber was heated to 650 °C for 12 min, and the sample was then exposed to C_2_H_4_ with a flow rate of 0.24 sccm for 40 min before it was cooled to RT in a vacuum. This approach reduces any oxidized TM back to a clean metallic surface before the growth of graphene.

**Figure 7:**
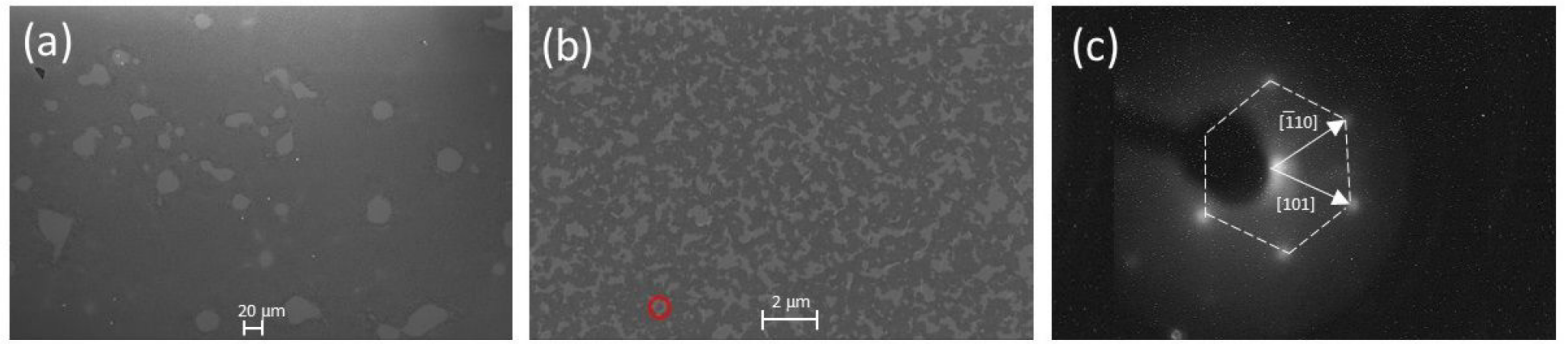
SEM images taken at 1 kV accelerating voltage showing the graphene domains for (a) epitaxial graphene/Ni and (b) rotated graphene/Ni. (c) The LEED diffraction pattern of epitaxial graphene on a Ni(111) substrate at 300 eV. The red circle in (b) highlights a single graphene domain with a diameter of ∼0.25 μm.

The SEM images shown in Figure 7 illustrate the structure of epitaxial and rotated graphene on Ni with a surface coverage of ∼70–90%. Figure 7a shows a homogeneous monolayer of graphene on Ni. The darker gray regions in Figure 7b are the differently oriented graphene domains, whereas the bright areas in Figure 7a and b are the bare Ni film. The low-energy electron diffraction (LEED) in Figure 7c shows the graphene’s hexagonal pattern epitaxially grown on the Ni(111) substrate. The (1 × 1) grown graphene structure is confirmed because no additional diffraction spots are observed in the LEED pattern.

### Raman Spectroscopy

Room-temperature Raman scans were taken at three different regions of each sample using a 532 nm excitation laser wavelength (50× objective lens and ∼1 μm laser spot size). Before the measurements, the graphene was first transferred by a chemical etching process from the metallic films onto a Si substrate with a 300 nm thermally oxidized SiO_2_ layer. This approach overcomes the fact that closely lattice-matched films lead to a loss in the resonance conditions for observing Raman spectra as a result of the strong chemical interaction between the graphene π orbital and the d-states of Ni and Ni_9_Mo_1_, which also alters the graphene’s p_*z*_ orbitals. Furthermore, the increase in the C–C bond length to match the lattice of the FM leads to significant changes in the graphene’s phonon spectrum.^23,65^ For the transfer process, the samples were cleaved into ∼5 mm × 5 mm squares, and HNO_3_, diluted to 5% for Ni_9_Mo_1_ and 10% for Ni, was used to etch the metallic films slowly while preserving the graphene layer.

### X-ray Magnetic Circular Dichroism (XMCD)

We carried out element selective XMCD measurements to detect and distinguish the magnetization in the graphene from that of the FM layer. The XMCD experiments were performed at 300 K at the SIM end station of the Swiss Light Source (SLS) at the Paul Scherrer Institut (PSI), Switzerland, using the total electron yield (TEY) detection mode with 100% circularly polarized light. The rotated-domain graphene/Ni sample was set to an incident angle of 30° from the incoming X-ray beam. An electromagnet was fixed at 40° to the incoming X-ray beam, and a magnetic field of 0.11 T was applied for 30 s in-plane to the surface of the samples to align the film magnetization along the beam direction. It was then reduced to 0.085 T during the X-ray absorption spectroscopy measurements. The intensity of the incident X-ray beam was measured with a clean, carbon free, gold mesh placed just before the sample position. This is particularly important for normalizing the signal at the C *K*-edge due to the presence of carbon on the surface of the X-ray optical components.

### Polarized Neutron Reflectivity

The PNR measurements were conducted at 10 and 300 K, under an in-plane magnetic field of 0.5 T, using the Polref instrument at ISIS spallation neutron source (UK). The fitting of the data was done using the *Refl1D*(66) software package with preliminary fits done in *GenX*.^67^ Although Ni is ferromagnetic at RT, the 10 K measurements are expected to provide a better estimation of the induced magnetic moment due to the lower thermal excitations of the electron spin at low temperature. Both the 10 and the 300 K data sets were fitted simultaneously to provide further constraint to the fits. This is analogous to the isotropic contrast matching^68,69^ used in soft matter neutron reflectivity experiments. This is very important in this case due to the attempt to measure a thin layer of graphene within a limited total *Q* range for PNR. The PNR is sensitive to only part of the broad fringe from the graphene layer, which acts as an envelope function on the higher frequency fringes from the thicker Ni layer underneath (the modeling methodology is discussed in detail in the Supporting Information).

## Supporting Information Available

Raman spectroscopy measurements for the graphene/Ni_9_Mo_9_ sample, summary of the Raman measurements of all of the samples, XMCD formulas used for the XMCD and XAS analyses presented in this Article, X-ray reflectivity data and the corresponding fit of the rotated graphene/Ni sample, and PNR modeling methodology describing the different scenarios considered for fitting the rotated graphene/Ni sample leading to model 16 presented in this Article (PDF)
