# Supplementary material for: Determining the Proximity Effect-Induced Magnetic Moment in Graphene by Polarized Neutron Reflectivity and X-ray Magnetic Circular Dichroism
Source: ACS Appl Mater Interfaces. 2023 Apr 24;15(18):22367–76. doi: 10.1021/acsami.2c02840 (PMC10176321; doi:10.1021/acsami.2c02840)
Supplement: Supplementary file 1 — am2c02840_si_001.pdf [file am2c02840_si_001.pdf]

Supplementary Material

for

Determining the Proximity Effect Induced Magnetic Moment in Graphene by Polarized Neutron Reflectivity and X-ray Magnetic Circular Dichroism

Razan O. M. Aboljadayel,[1, a)] Christy J. Kinane,[2] Carlos A. F. Vaz,[3] David M. Love,[1] Robert S. Weatherup,[4] Philipp Braeuninger-Weimer,[4] Marie-Blandine Martin,[4] Adrian Ionescu,[1] Andrew J. Caruana,[2] Timothy R. Charlton,[2] Justin Llandro,[1] Pedro M. S. Monteiro,[1] Crispin H. W. Barnes,[1] Stephan Hofmann,[4] and Sean Langridge[2, b)]

[1)] *Cavendish Laboratory, Physics Department, University of Cambridge, Cambridge CB3 0HE, United Kingdom.*

[2)] *ISIS Facility, STFC Rutherford Appleton Laboratory, Harwell Science and Innovation Campus, Oxon, OX11 0QX, United Kingdom.*

[3)] *Swiss Light Source, Paul Scherrer Institut, 5232 Villigen PSI, Switzerland*

[4)] *Department of Engineering, University of Cambridge, Cambridge CB3 0FA, United Kingdom.*

S1. RAMAN MEASUREMENTS FOR THE GRAPHENE/Ni_9Mo_1 SAMPLE

Graphene has two main characteristic peaks in the Raman spectra, a first-order Raman scattering (RS) G peak at $\sim$ 1582 cm^{-1}, which is a graphite-like line that can be observed in different carbon-based materials;[1–3] and a second-order (double-resonance) RS $2D$ peak at $\sim$ 2700 cm^{-1}, which is usually used as an indication of a perfect crystalline honeycomb-like structure.[1,3] Graphene also possesses other second-order RS peaks such as D+D'' located at $\sim$ 2450 cm^{-1} and $2D'$ at $\sim$ 3200 cm^{-1} and disorder-induced peaks such as D at $\sim$ 1350 cm^{-1} and D' at $\sim$ 1600 cm^{-1}.[3,4]

Single-layer graphene is known to have three characteristic features; I_{2D}/I_G ratio > 2 and a $2D$ peak fitted with a single Lorentzian with a full-width at half-maximum (FWHM) less than 40 cm^{-1}.[5] Furthermore, I_D/I_G is usually used as an indicator of defects present in graphene, which increases with the increase of disorder in the graphene structure.[1,5] Therefore, good-quality graphene possesses a small I_D/I_G ratio. The D peak was reported to change in shape, position and width by increasing the number of graphene layers. Also, the G peak is known to downshift with increasing the number of graphene layers but upshift with increasing the doping level.[3] Moreover, the width of the $2D$ peak increases with the number of graphene layers.[4,6–8] Therefore, we assess the quality of our transferred graphene based on these features. Figure S1 shows the room temperature (RT) Raman spectra for graphene transferred from the Ni_9Mo_1 film. The average peak positions and the average $2D$ FWHM of all the samples are listed in Table S1.

The Raman spectra of the transferred graphene from Ni_9Mo_1 (Figure S1 (a)) show a high $I_{D'}$. Therefore, an argument similar to that used for the graphene transferred from Ni(111) can be applied here to explain the spectra. However, the wide spatial variation across the surface of the sample, which could be attributed to the formation of occasional strong Mo−C bonding between the graphene and the Ni_9Mo_1 film, makes it difficult to assess the quality of the graphene grown on Ni_9Mo_1 based on the $2D$ FWHM.

S2. X-RAY MAGNETIC CIRCULAR DICHROISM (XMCD) FORMULAE

The X-ray absorption amplitude (μ_{XAS}) and the X-ray magnetic circular dichroism (μ_{XMCD}) are expressed as:

$$\mu_{\mathrm{XAS}} = \frac{1}{2}(\mu_+ + \mu_-), \quad (1)$$

and

$$\mu_{\mathrm{XMCD}} = \mu_+ - \mu_-. \quad (2)$$

μ_+ and μ_- are the absorption coefficients for the right and left circularly polarized light, respectively, normalized to a common value.[9]

According to the sum rules, XAS and XMCD spectra at the $L_{2,3}$-edge can be used to determine the $3d$ orbital angular momentum $< L_z >$ and the spin angular momentum $< S_z >$ using the following expressions:[10,11]

$$< L_z > = -2n_h \cdot \frac{\int_{L_3+L_2}(\mu_+ - \mu_-)dE}{\int_{L_3+L_2}(\mu_+ + \mu_- + \mu_{XAS})dE}, \quad (3)$$

[a)] Electronic mail: roma2@cantab.ac.uk
[b)] Electronic mail: sean.langridge@stfc.ac.uk

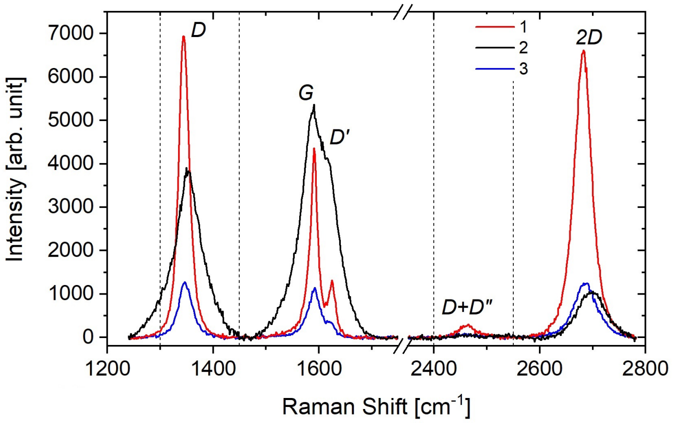

FIG. S1. Room temperature Raman spectroscopy measurements for graphene transferred from the $Ni_9Mo_1(111)$ film taken at three different regions, showing the graphene's characteristic peaks. The dashed vertical lines indicate regions for the different peaks.

TABLE S1. Results of the Raman measurements summarizing the average peaks positions and the 2*D* average FWHM for graphene transferred from rotated Ni, epitaxial Ni and Ni_9Mo_1 films.

Sample	*D* [cm^{-1}]	*G* [cm^{-1}]	*D'* [cm^{-1}]	*D*+*D''* [cm^{-1}]	2*D* [cm^{-1}]	2*D* FWHM [cm^{-1}]
Rotated Ni(111)	1346.3	1590.7	1624.8	2464.2	2681.9	46.2
Epitaxial Ni(111)	1344.6	1590.7	1623.1	2462.6	2682.6	40.8
$Ni_9Mo_1(111)$	1346.3	1590.7	1619.9	2464.0	2682.6	39.6

$$< S_z >= -\frac{3}{2}n_h \cdot \frac{\int_{L_3}(\mu_+ - \mu_-)dE - 2\int_{L_2}(\mu_+ - \mu_-)dE}{\int_{L_3+L_2}(\mu_+ + \mu_- + \mu_{XAS})dE} \cdot \left(1 + \frac{7 < T_z >}{2 < S_z >}\right). \quad (4)$$

Here, $n_h = (10 - n_d)$ is the number of holes in the 3*d* states, whereas n_d is the electron occupation number of the 3*d* states. $L_3 + L_2$ represent the integration range over the L_3 and L_2 edges and $< T_z >$, which is the expectation value of the magnetic dipole operator, is known to be very small in TMs and hence it was ignored.[12] In (2), $\mu_{\rm XMCD}$ is corrected for the light degree of polarization, *P*, and the light's incident angle, θ. Therefore, it was multiplied by a factor $\frac{1}{P\cos\theta}$,[13] where θ is measured with respect to the sample's surface, while $\mu_{\rm XAS}$ remains unchanged.[14]

The orbital magnetic moment, m_o, is equal to $\mu_{\rm B} < L_z >$, whereas the spin magnetic moment, m_s, is given by $m_s = 2\mu_{\rm B} < S_z >$.[11] For the total magnetic moment, $m_{total} = m_o + m_s$.[11,15,16]

In contrast, since the XMCD at the *K*-edge measures the transitions from a non-spin-split *s* orbital to *p* orbital, only m_o can be obtained:[17,18]

$$m_o = -\frac{1}{3}\frac{n_h}{P\cos\theta}\frac{\int_K \mu_{XMCD}}{\int_K \mu_{XAS}}, \quad (5)$$

For the *K*-edge, n_h becomes equal to $6 - n_p$, where n_p is the electron occupation number in the 2*p* bands.[18] Since $m_s = 2m_o/(g-2)$, where *g* is the gyromagnetic factor,[19] m_{total} can be estimated for the *K*-edge if *g* is known.

S3. X-RAY REFLECTIVITY

An X-ray reflectivity scan was performed on the rotated graphene sample using a 9 kW rotating anode Rigaku SmartLab X-ray diffractometer, with Cu K_α radiation with a wavelength of 1.541 Å (Figure S2). These data were initially difficult to analyse due to the fit being dominated by the extremely good statistics at low *Q* around the critical edge. This is due to the problem that the Poisson error bars are smaller than the footprint correction.[20,21] The line shape from the footprint distorts the data and the initial fit could not get past the critical edge and fit the rest of the film, which resulted in χ^2 values in the many thousands. While it was possible to apply a simple footprint correction, we noted that the sample length and beam size parameters required were not only correlated to each other, but also correlated to the densities and roughnesses in the fit, leading to non-quantitative results. In order to minimise this problem, we applied a footprint correction to the data using the Reductus package[22,23] yielding the fit shown in Figure S2(a) with $\chi^2 = 4$. However, there are still outstanding issues with the data, as indicated by the blue circles in the figure, and these are extremely difficult to solve. We could link these issues again to the footprint correction, which does not allow to generate a good fit or provides the possibility of co-fitting the XRR with the neutron results. We direct the interested reader to the Open Reflectivity Standards Organisation (ORSO) group[24] seminar on this topic[25] for a detailed discussion. These issues become again evident in the residuals trace plotted in panel (b) of Figure S2.

We would like to point out that Time of Flight (ToF) reflectometry method used for the polarized neutron reflectivity

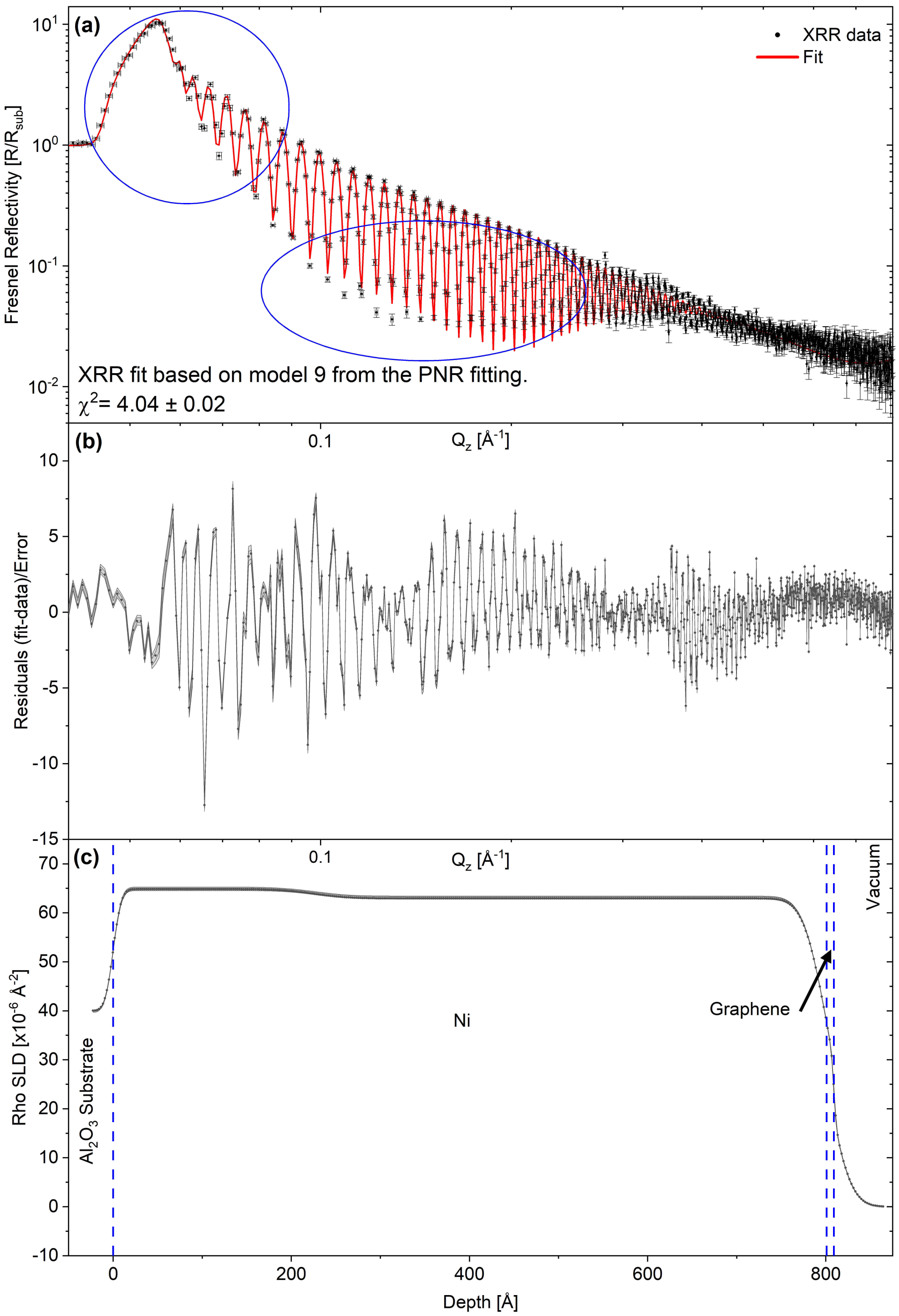

FIG. S2. XRR: Model based on model 9 from the PNR fitting, where the Ni layer is divided into two, with a graphene layer on the surface. The graphene layer thickness is constrained to be equal or thicker than 2 monolayers of graphene (8.1Å). (a) X-ray Fresnel reflectivity (b) Residuals for the XRR fit (c) Electron scattering length density profile. The grey banded regions around the SLD lines are the 95% Bayesian confidence intervals.

data does not suffer from the same problems with geometric footprint corrections as monochromatic reflectivity due to the nature of the ToF technique. The best fit to the XRR data was obtained using a model similar to model 9 from the PNR fit-

ting. The thickness of the Ni layer was nominally the same as the intended growth thickness of ≈ 800 Å. However, the XRR technique is less sensitive to the graphene layer due to the low electron density of C compared to the Ni. This meant that we had to limit the graphene thickness to a minimum thickness of two monolayers in agreement with the SEM and Raman data. What was qualitatively reproduced was that a graded Ni layer density was required to get a good fit, as shown in panel (c) of Figure S2. These thicknesses and roughnesses were then used as initial starting points for the PNR modeling.

S4. POLARIZED NEUTRON REFLECTOMETRY ANALYSIS

The primary analysis of the PNR data was performed using the *Refl1D* software package from the National Center for Neutron Research (NCNR) at the National Institute for Standards and Technology (NIST).[26] *Refl1D* uses the Bayesian analysis fitting package Bumps,[27] for both obtaining the best fit and performing an uncertainty analysis on the parameters used to model the data. *Refl1D* is based on the Abeles optical matrix method,[28] along with interfaces approximated using the method of Névot and Croce.[29] *Refl1D* allows multiple data sets to be fitted simultaneously, which is advantageous in this case due to the difficulty of detecting such a thin layer of graphene on top of a thick layer of Ni metal. Hence the data taken at the two temperatures (10 K and 300 K) were fitted simultaneously for each sample to reduce the uncertainty in the fitted parameters, such as the magnetic contrast variation. This is akin to the isotopic contrast variation method used as standard in non-polarized neutron reflectivity soft matter experiments.[30]

A. Modeling methodology

The analysis was started on the rotated-domain graphene/Ni sample using the simplest model possible as a starting point. Once convergence was reached as defined in the *Refl1D* documentation,[31] an assessment was made taking into account the improvement of the χ^2 and the uncertainty in the parameters as to whether to increase the complexity of the model by adding more parameters. Prior knowledge of the sample growth conditions and sample characterisation was also employed to aid in this process. We echo what is stated in the *Refl1D* documentation[31] that, since this is a parametric modeling technique, we cannot say that a model is truly correct, only that while it fits the observed data well, there may be other possible solutions. We couple this argument with the following points to analyse the reflectivity:

- A well-defined question for what the model is intended to answer. This provides a measure of how well the model is working, but most importantly, a point at which to stop fitting the data. It can also be instrumental in determining if the data quality is insufficient to answer the posed question. Having a well-defined analysis question, such as whether a particular layer is magnetic or how thick the surface layer is, is crucial for any reflectivity analysis.
- Knowledge of the sample growth conditions to provide, initial priors (maximum and minimum bounds on those initial parameters) and a nominal starting structure for the fitting model. In particular, the substrate material parameters should be well known.
- Magnetic contrast variation, which is akin to solving simultaneous equations. The soft matter community makes great use of contrast variation, both magnetic contrast and, more commonly, nuclear contrast, by swapping out hydrogen for deuterium atoms.[30] This greatly aids them in reducing the parameter search space and improves the uncertainty in their results.
- Secondary characterization techniques, which provide crucial information that can be used as a cross-reference for particular parameters to lock them down, e.g. SQUID for the total magnetic moment of the sample. This also becomes important when dealing with issues arising from the inverse phase problem, such as multiple solutions with similar χ^2 value, i.e. multi-modal fit solutions. Examples of this point are given later in this SI. Furthermore, these techniques are also invaluable for setting priors.

Such information provides confidence in the validity of the final model and avoids over-parameterization.

In the present case the questions are: Can the model confirm that there is a graphene layer present and primarily is it magnetic? Can the magnitude of the magnetic moment be measured with any certainty?

Considering all the above points makes it possible to construct a whole series of models and compare and contrast them, allowing us to answer the stated questions confidently.

So to start the process, the initial model should be the simplest model possible. It is taken as given that this should always be the first model run in any reflectivity analysis. The aim is to provide a null result to test against it to see if the graphene is required to fit the data set.

Model 1 consists of a single layer of Ni on an Al_2O_3 substrate with the Ni magnetism commensurate with the boundaries of the Ni Layer. There are no magnetic dead layers or graphene included.

Knowledge from the sample growth, along with the XRR, SEM and Raman spectroscopy scans, were used to inform the initial starting guesses for all parameters and, most importantly, the prior (Max/Min) fit ranges. All priors were flat in probability distribution.[32]

For the starting guess, the Ni thickness, where the nominal growth thickness was intended to be 800 Å, which was confirmed (within 5%) by the XRR modeling, was allowed to vary by 10% for a prior fit range. The physical density of the Ni was taken from the NIST database for neutron scattering lengths and cross sections.[33] For models that include

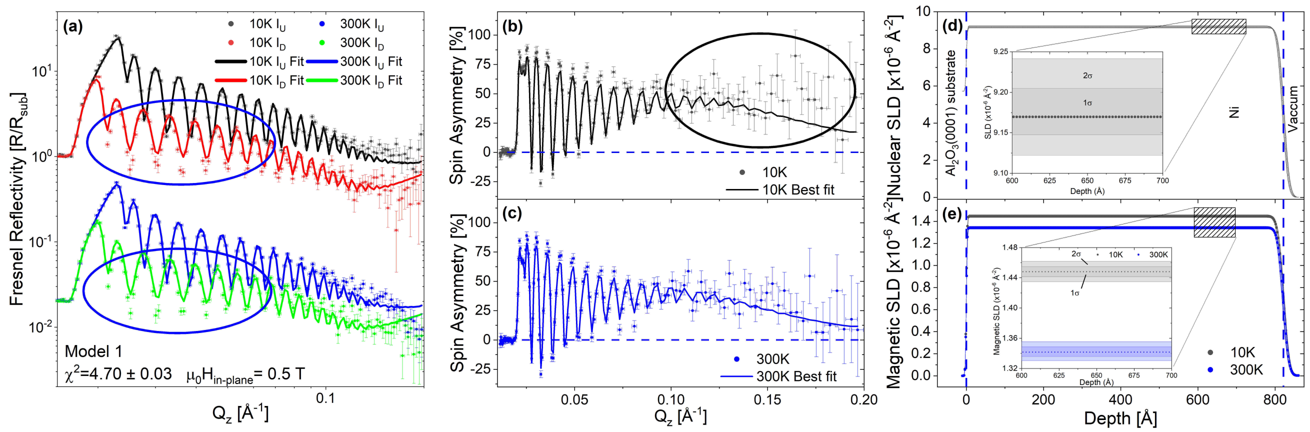

FIG. S3. PNR Model 1: Single Ni layer with commensurate magnetism. (a) Fresnel reflectivity for 10 K and 300 K, (b) and (c) show the spin asymmetries, (d) is the nuclear scattering length density (nSLD) profile and (e) the magnetic scattering length density (mSLD) profiles. The grey banded regions around the SLD lines are the 95% Bayesian confidence intervals. The room temperature Fresnel reflectivity has been shifted by a factor of 50 for ease of display.

graphene, a density of 2.267 g/cm^2 was used.[34] However, this was then allowed to vary by ±25%, as the density is ill-defined depending on the graphene environment and its quality.[34] Furthermore, the graphene thickness was known to be between 2 and 4 monolayers from the SEM and Raman scans. Therefore, the maximum prior was set initially to $12-15$ Å (i.e. approximately 4 monolayers) with a 2 Å lower limit. The Ni magnetic moment was initially taken as 0.6 μ_B/atom (508.6 emu/cm^3, giving approximately a Ni mSLD of 1.454 x 10^{-6} Å^{-2}),[35] and it was used as the maximum positive and maximum negative priors so the magnetic moment could vary through zero. The graphene magnetic moment was set to vary by the same amount as the Ni as the XMCD results indicate similar magnetism in both layers. A nominal density for Al_2O_3 of 3.98 g/cm^2 was used for the substrate and kept constant.[36] The roughnesses of all layers were initially set to 5 Å and then they were allowed to vary from 2 Å to 50% of each layer's starting thickness. However, for the substrate, this was set to a maximum of 50 Å. These prior ranges were then adjusted as needed, as described below.

The results for Model 1 are shown in Figure S3. Panel (a) displays the Fresnel reflectively ($R_{Fresnel}$) given by:

$$R_{Fresnel} = \frac{R}{R_{Substrate}}, \quad (6)$$

which is the total reflectivity (R) divided by the calculated substrate reflectivity ($R_{Substrate}$). $R_{Fresnel}$ is plotted as it visually aids in finding discrepancies between the fit and the data. It is evident that the fit produced by this model does not match the depth of the fringes at low Q, as indicated by the blue circle.

The spin asymmetries (*SA*) of the 10 K and 300 K are presented in panels (b) and (c), respectively. The results show that the fit does not match the *SA* data at high Q in the same regions where the $R_{Fresnel}$ curves up at high Q. This indicates a thin and magnetic layer is missing from the model, such as a thin graphene layer. Furthermore, the oscillations don't match at low Q. Panel (d) shows the nuclear scattering length density (nSLD) profile. The insets show the 68% and 95% Bayesian confidence intervals from the Bumps fitting package, which gives an indication of how certain the model is with regard to the fit of the data. However, we stress that this only shows how well the current model fits the data, it does not confirm if the model is the correct one or not.

It follows from model 1 that a thin layer or layers is required to fit the high Q data. This is not necessarily a graphene layer, it could be a thin magnetic dead layer. Therefore we constructed model 2 shown in Figure S4. This model consists of a single layer of Ni on an Al_2O_3 substrate where the Ni magnetism is now incommensurate with the boundaries of the Ni layer, i.e. we allow Ni dead layers with a moment that can vary from zero to the full Ni moment, and have thicknesses and roughnesses independent of the nSLD profile. Although this is intended as a null case, it slightly improves the χ^2 however still fails to fit the low and high Q regions that model 1 did not reproduce.

Model 3, shown in Figure S5, consists of a single layer of Ni on an Al_2O_3 substrate with the Ni magnetism commensurate with the boundaries of the Ni layer and a non-magnetic graphene layer on the top surface. This model produces a fit with a similar χ^2 value to model 2 that still fails to sort the issues from the previous models at high and low Q ranges.

The next step (model 4) is to allow the graphene layer to be magnetic as shown in Figure S6, while keeping the rest of the model the same as model 3. Although this produces a small step change in the χ^2 value where the high Q data is significantly improved, it does not capture the low Q fringes. However, it provides evidence that we need the graphene layer to be magnetic to fit the data. At this point it seems likely that some combination of Ni dead layers and magnetic graphene

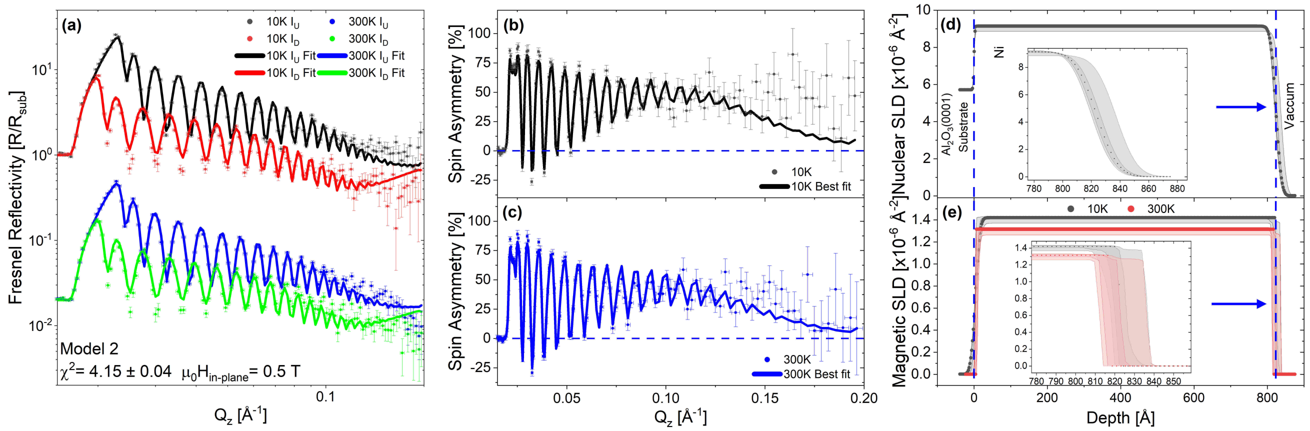

FIG. S4. PNR Model 2: Ni layer with dead layers top and bottom. (a) Fresnel reflectivity for 10 K and 300 K, (b) and (c) show the spin asymmetries, (d) is the nuclear scattering length density (nSLD) profile and (e) the magnetic scattering length density (mSLD) profiles. The grey banded regions around the SLD lines are the 95% Bayesian confidence intervals. The room temperature Fresnel reflectivity has been shifted by a factor of 50 for ease of display.

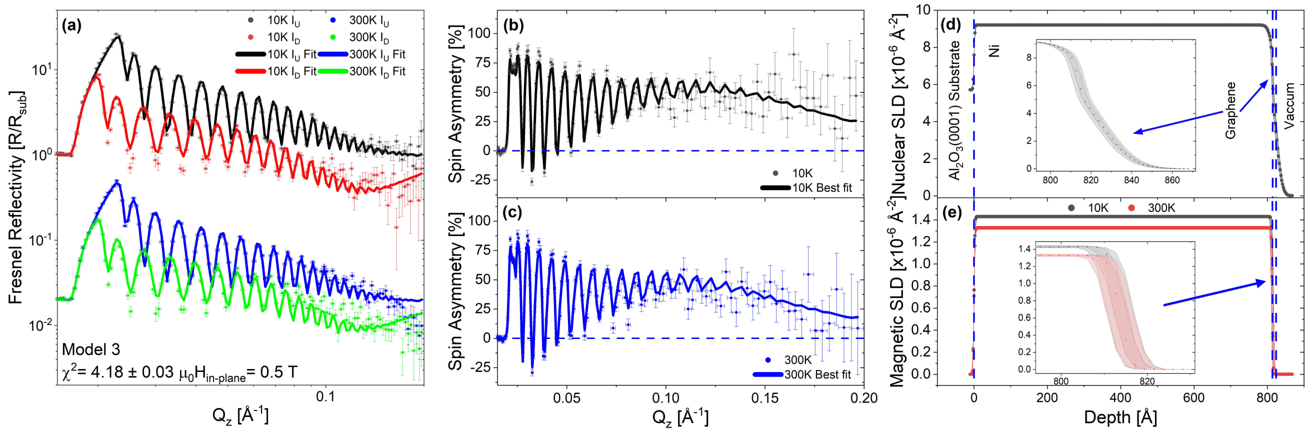

FIG. S5. PNR Model 3: Ni layer with commensurate magnetism and a non-magnetic graphene layer on the surface. (a) Fresnel reflectivity for 10 K and 300 K, (b) and (c) show the spin asymmetries, (d) is the nuclear scattering length density (nSLD) profile and (e) the magnetic scattering length density (mSLD) profiles. The grey banded regions around the SLD lines are the 95% Bayesian confidence intervals. The room temperature Fresnel reflectivity has been shifted by a factor of 50 for ease of display.

maybe at play, however, certainty on the values eludes us since the fit cannot reproduce the low Q data.

Model 5, shown in Figure S7, is another null model, where the statistics dominate, consisting of a single layer of Ni on an Al_2O_3 substrate with the Ni magnetism having dead layers at the top and bottom with a non-magnetic graphene layer at the surface. This model yields a significant improvement in χ^2, as expected from the earlier model, hinting that some combination is required.

Model 6 takes model 5 and allows the graphene to be magnetic. This results in a magnetic discrepancy at the top interface due to a small region of magnetically dead Ni layer leading to magnetic graphene, as shown in the mSLD in Figure S8 (e). This also manifests as a spike in the magnetization of the graphene layer and is not physical.

Model 7, presented in Figure S9, removes the top Ni dead layer allowing continuous magnetism into the graphene layer. Although this model does not improve the χ^2 value over model 6, it slightly improves the top interface. However, there are still issues highlighted by red circles shown in panels (d) and (e), where this time the Ni roughness violates the Névot-Croce[29] approximation and exceeds the thickness of the graphene. This produces non-physical magnetic profiles with a peak and dip at the interface regions, which is also present in Model 6. It is clear that the fit is limited by the low Q features indicated by the blue circles in Figure S9 (a). Therefore, the fit cannot be improved at the high Q range unless the low Q is improved due to the weighting of the statistics.

It should be noted that the *Refl1D* package allows for a full

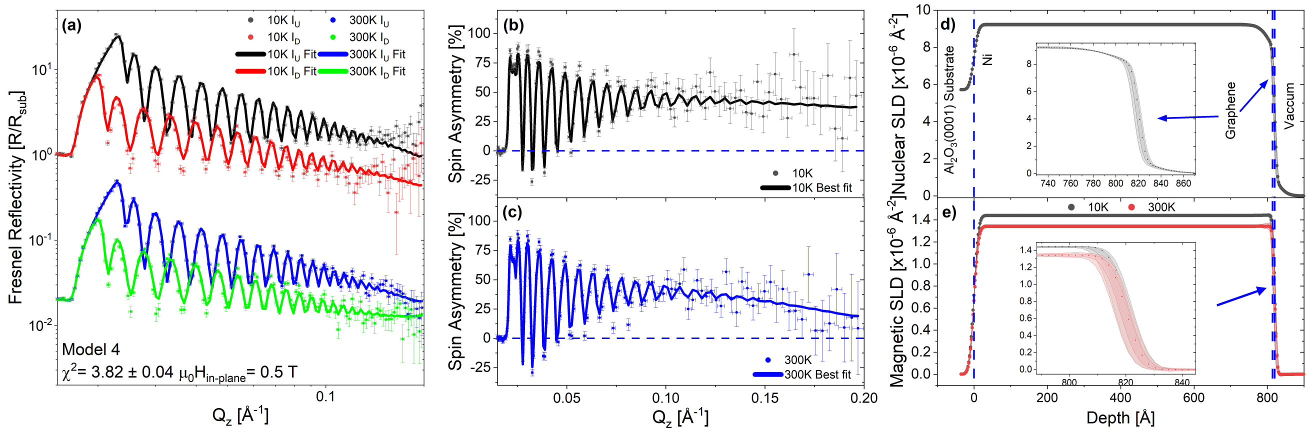

FIG. S6. PNR Model 4: Ni layer with commensurate magnetism and a magnetic graphene layer on the surface. (a) Fresnel reflectivity for 10 K and 300 K, (b) and (c) show the spin asymmetries, (d) is the nuclear scattering length density (nSLD) profile and (e) the magnetic scattering length density (mSLD) profiles. The grey banded regions around the SLD lines are the 95% Bayesian confidence intervals. The room temperature Fresnel reflectivity has been shifted by a factor of 50 for ease of display.

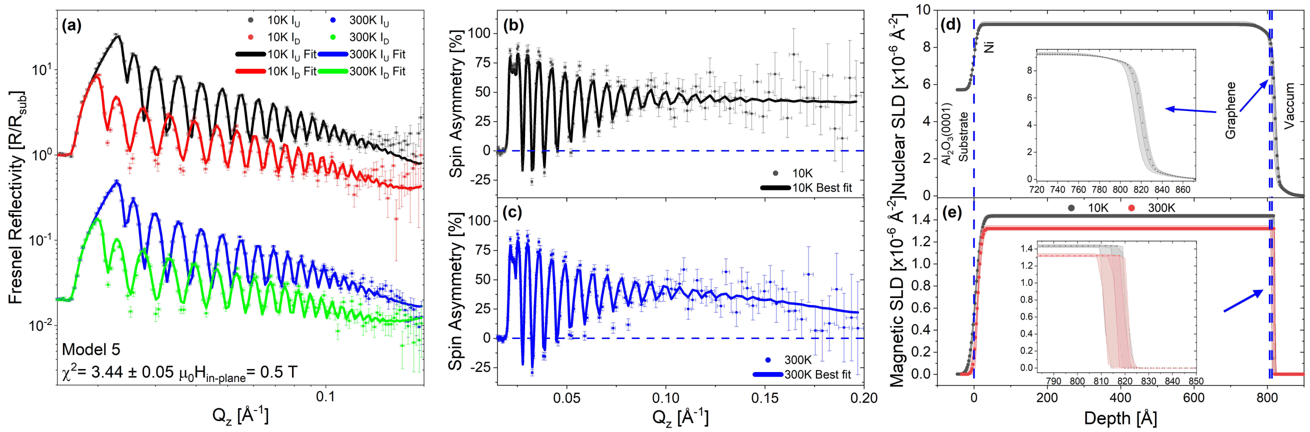

FIG. S7. PNR Model 5: Ni layer with dead layers top and bottom a non-magnetic graphene layer on the surface. (a) Fresnel reflectivity for 10 K and 300 K, (b) and (c) show the spin asymmetries, (d) is the nuclear scattering length density (nSLD) profile and (e) the magnetic scattering length density (mSLD) profiles. The grey banded regions around the SLD lines are the 95% Bayesian confidence intervals. The room temperature Fresnel reflectivity has been shifted by a factor of 50 for ease of display.

matrix calculation, dispensing with the Névot-Croce approximation, and slicing up the interfaces, which was done with 2 Å slices. However, this dramatically increased the time for the analysis to converge, while a little difference was found between the two. Therefore, the decision was taken to keep the Névot-Croce approximation for the interfaces to save computational time.

Models $1-7$ show that small changes to the graphene and magnetic dead layers have no effect on the low Q oscillations whose statistics are dominating the χ^2 and so prevent a good fit of the high Q. It is noteworthy to mention that high Q fringes give information about the graphene layer, whereas that at low Q range provide details about the Ni film. Model 8, shown in Figure S10, takes model 7 and splits the Ni layer into two, allowing a large roughness between the two layers to grade the nSLD and mSLD, while the two are kept magnetically linked. Having two very similar nSLD layers allows some extra contrast and beat frequencies that can modify the amplitude of the Kiessig oscillations. This dramatically improves the χ^2 and the uncertainties on the graphene layer as the fit is no longer dominated by the residuals at low Q. We have little justification for this, but as the Ni layer is relatively thick, if there is epitaxy to the Al_2O_3 substrate then the approximately 80 nm Ni(111) layer may relax its stress/strain across this thickness, producing an nSLD gradient across the layer. This also has an almost mirror image effect on the mSLD profile at 300 K.

Model 8 is a workable fit demonstrating magnetism in the graphene layer, however the graphene layer thickness reduces down to $\sim$ 2 Å, which is right at the fitting limit set for

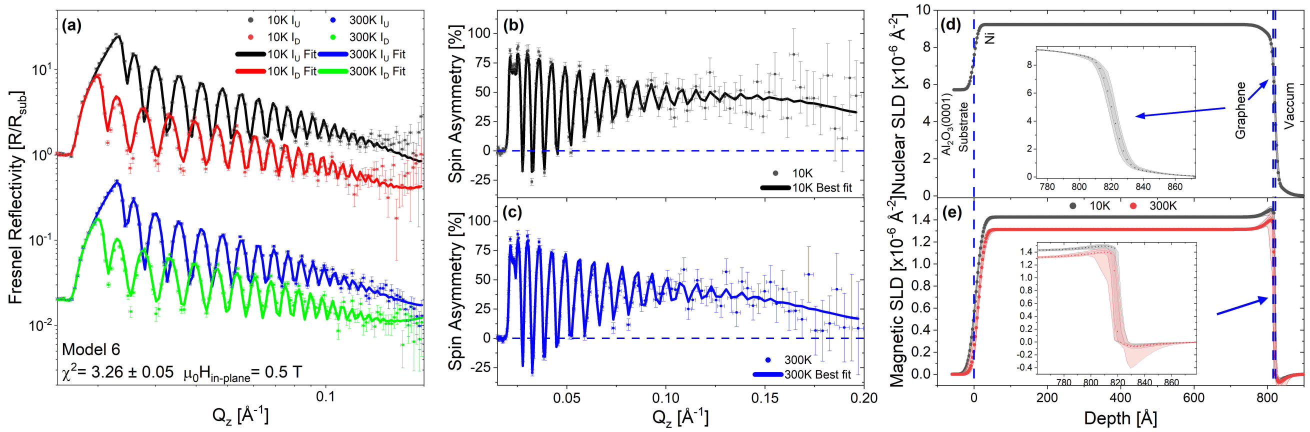

FIG. S8. PNR Model 6: Ni layer with dead layers top and bottom and a magnetic graphene layer on the surface. (a) Fresnel reflectivity for 10 K and 300 K, (b) and (c) show the spin asymmetries, (d) is the nuclear scattering length density (nSLD) profile and (e) the magnetic scattering length density (mSLD) profiles. The grey banded regions around the SLD lines are the 95% Bayesian confidence intervals. The room temperature Fresnel reflectivity has been shifted by a factor of 50 for ease of display.

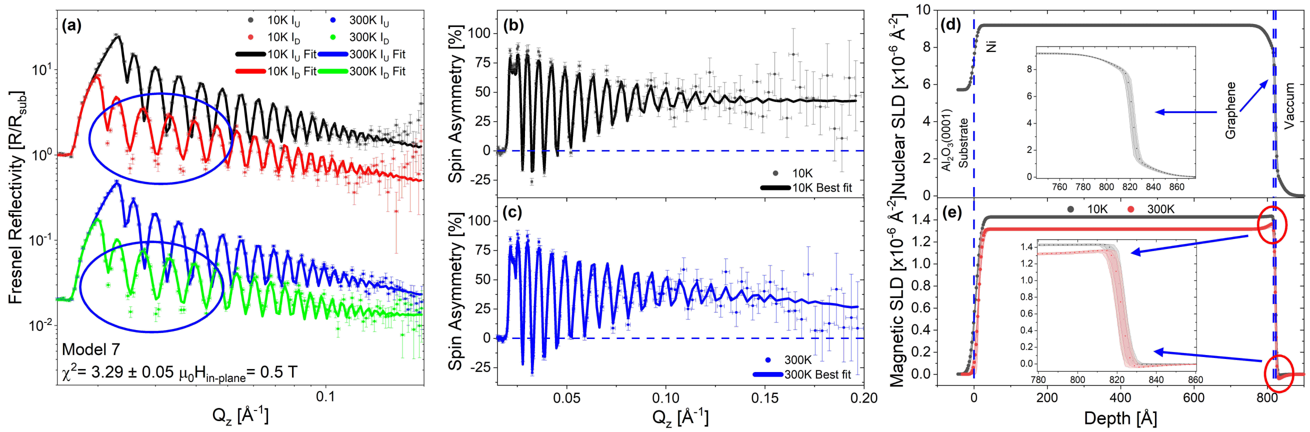

FIG. S9. PNR Model 7: Ni layer with a dead layer at the substrate interface and continuous magnetism into a magnetic graphene layer on the surface. (a) Fresnel reflectivity for 10 K and 300 K, (b) and (c) show the spin asymmetries, (d) is the nuclear scattering length density (nSLD) profile and (e) the magnetic scattering length density (mSLD) profiles. The grey banded regions around the SLD lines are the 95% Bayesian confidence intervals. The room temperature Fresnel reflectivity has been shifted by a factor of 50 for ease of display.

one monolayer. The uncertainty in the fitted parameter indicates that we are not very sensitive to it. At this point we have not included any information from other characterization techniques. Both SEM and Raman spectroscopy measurements suggest that the rotated-domain graphene/Ni(111) sample should have approximately two to three monolayers of graphene on the surface. Therefore, if we use this information to set the lower bound on the prior for the graphene thickness, we get model 9 (Figure S12) which produces a fit within the error of the χ^2 value of model 8 but utilises all the available information.

To further demonstrate the validity of this choice, we obtain the correlation plots between parameters from the *Refl1D* and nested sampler software packages. Although there were several bi-modal correlations in Model 8 and 9's fits for the graphene thickness and the graphene and top Ni interface roughnesses, only some of the cleanest examples from model 9 are presented in Figure S13. The plots show how crucial the inclusion of secondary information is in determining the best model, as it effectively allows the selection of the right node. In our case, 20 to 25 Å of graphene is equivalent to $4-5$ monolayers, but the SEM and Raman suggest that we only have 2 to 3 monolayers. Hence limiting the graphene thickness to 2 to 3 monolayers is a valid choice.

Four further checks were performed to study the sensitivity of the PNR modeling to the magnetic moment in the graphene layer. This is now a crucial check as all the fits discussed previously (i.e. before model 9) have been dominated by the residuals at low Q. These were not adequately modeled until the Ni layer was allowed to be graded. Therefore, model 10

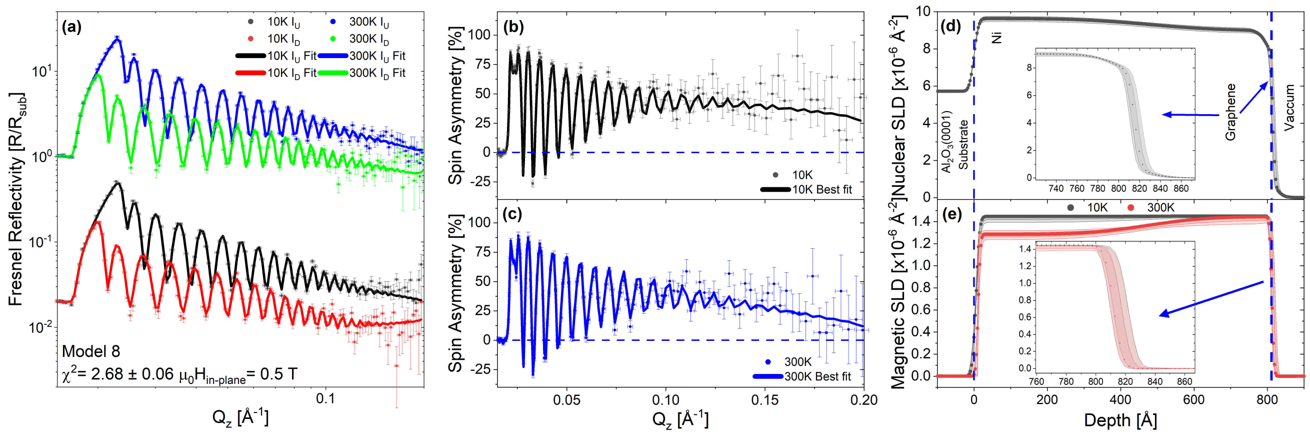

FIG. S10. PNR Model 8: Ni layer with a dead layer at the substrate interface and continuous magnetism into a magnetic graphene layer on the surface. Graphene layer thickness is unconstrained. (a) Fresnel reflectivity for 10 K and 300 K, (b) and (c) show the spin asymmetries, (d) is the nuclear scattering length density (nSLD) profile and (e) the magnetic scattering length density (mSLD) profiles. The grey banded regions around the SLD lines are the 95% Bayesian confidence intervals. The room temperature Fresnel reflectivity has been shifted by a factor of 50 for ease of display.

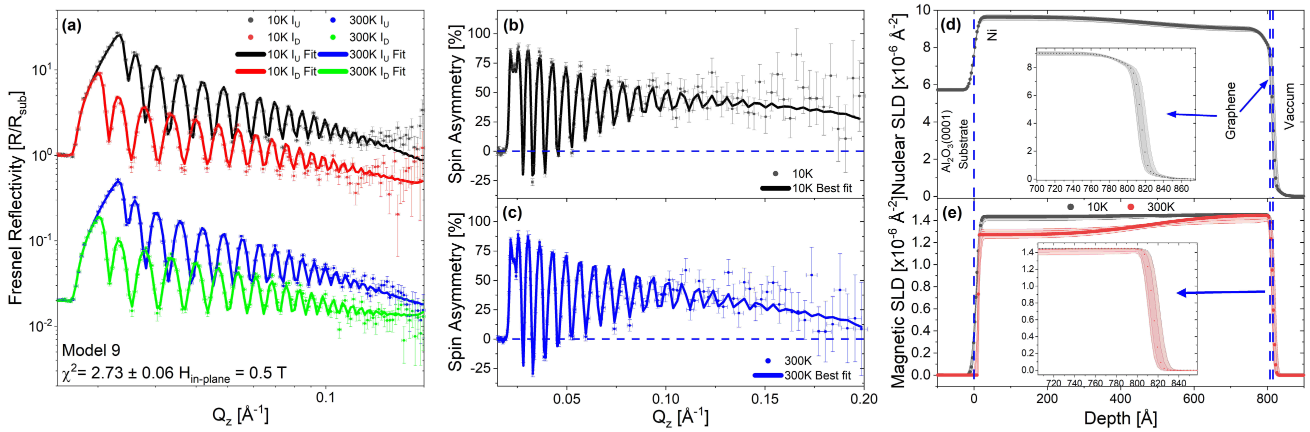

FIG. S11. PNR Model 9: Ni layer with a dead layer at the substrate interface and continuous magnetism into a magnetic graphene layer on the surface. The graphene layer thickness is constrained to be thicker than 2 monolayers of graphene (8.1 Å). (a) Fresnel reflectivity for 10 K and 300 K, (b) and (c) show the spin asymmetries, (d) is the nuclear scattering length density (nSLD) profile and (e) the magnetic scattering length density (mSLD) profiles. The grey banded regions around the SLD lines are the 95% Bayesian confidence intervals. The room temperature Fresnel reflectivity has been shifted by a factor of 50 for ease of display.

(Figure S12), is based on model 9, but the magnetic moment in graphene is fixed to zero. The fit produced a χ^2 only slightly worse than model 9 see Table S2. This indicates we are still a little sensitive to the magnetism in graphene.

The next test (model 11) is where the graphene layer is removed entirely, the results of which are shown in Figure S14. Again, the magnetism in the Ni has dead layers on top and bottom. Surprisingly, this almost matches Model 9's χ^2, being fractionally worse but with overlapping error bars.

Models 12 and 13 shown in Figures S15 and S16 are copies of models 10 and 11, respectively, but with dead layers at the bottom interfaces only. The magnetism at the top is conformal with the nuclear structure as in model 9. Both produce worse fits than model 9 and lend weight to the idea that either a graphene layer or a top magnetic dead layer in the Ni is needed to improve the fit.

We have mentioned previously that we are concerned about the violation of the Névot-Croce approximation,[29] where the roughness of a layer becomes larger than its thickness. This is clearly the case for some of the earlier models, with the thin graphene layer often becoming thinner than its roughness and that of the Ni layer directly below it. Until this point, we have endeavoured to prevent this from happening and have ensured that the graphene roughness can never exceed the graphene layer thickness via setting relevant priors (max/min fitting ranges with a flat probability profile as most analysis packages have by default).

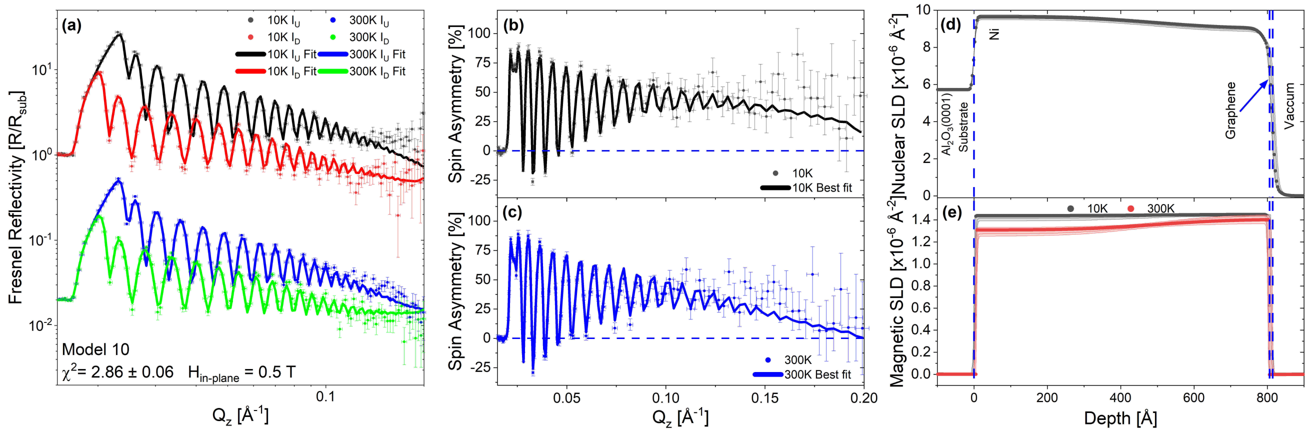

FIG. S12. PNR Model 10: Ni layer with a dead layer at the substrate interface and continuous magnetism into a magnetic graphene layer on the surface. The Ni has been split into two continuous Ni layers to allow a gradient across the layer. The graphene layer is constrained so it cannot be thinner than 2 monolayers of graphene in line with the SEM and Raman results. It has also been set to be non-magnetic. (a) Fresnel reflectivity for 10 K and 300 K, (b) and (c) show the spin asymmetries, (d) is the nuclear scattering length density (nSLD) profile and (e) the magnetic scattering length density (mSLD) profiles. The grey banded regions around the SLD lines are the 95% Bayesian confidence intervals. The room temperature Fresnel reflectivity has been shifted by a factor of 50 for ease of display.

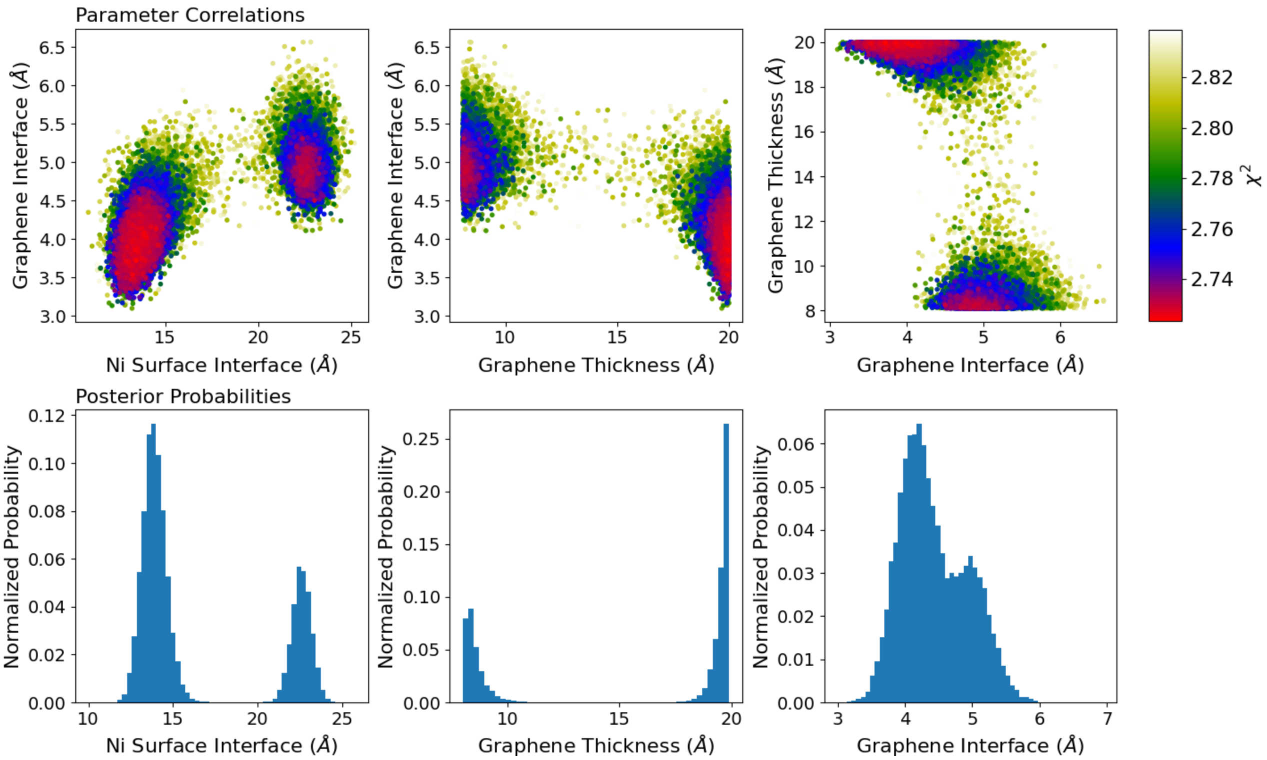

FIG. S13. Correlation plots and posterior probability distributions for the parameters exhibiting bi-modal behaviour from Model 9. Secondary information (obtained from the SEM and Raman spectroscopy measurements) was used to select one mode as preferable in each case.

Due to the thin nature of graphene, it is hard to envisage a set of physical circumstances where this can hold true. This is because the graphene sits on a Ni surface that is an order of magnitude rougher than the thickness of the graphene. Therefore, it is more likely that the graphene layer is conformal to the roughness of the underlying Ni film. This is analogous to the tarmac of a road following the undulations of a set of hills over which it traverses. In this scenario, it holds that the roughness of the graphene will be larger than its thickness as it follows the contours of the Ni hills it sits upon and must therefore violate the Névot-Croce approximation.

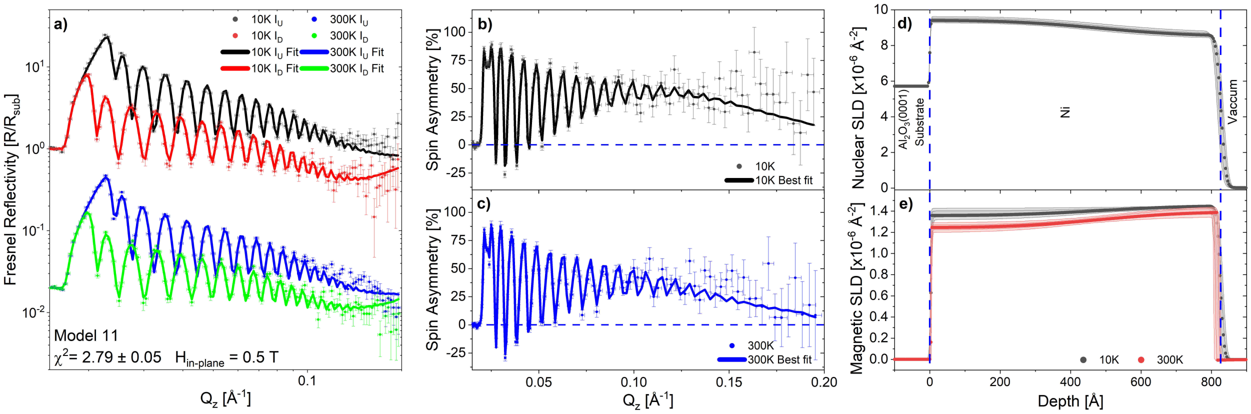

FIG. S14. PNR Model 11: Ni layer has dead layers at the bottom and top Ni interfaces. The Ni has been split into two continuous Ni layers to allow a gradient across the layer and the graphene layer has been removed. (a) Fresnel reflectivity for 10 K and 300 K, (b) and (c) show the spin asymmetries, (d) is the nuclear scattering length density (nSLD) profile and (e) the magnetic scattering length density (mSLD) profiles. The grey banded regions around the SLD lines are the 95% Bayesian confidence intervals. The room temperature Fresnel reflectivity has been shifted by a factor of 50 for ease of display.

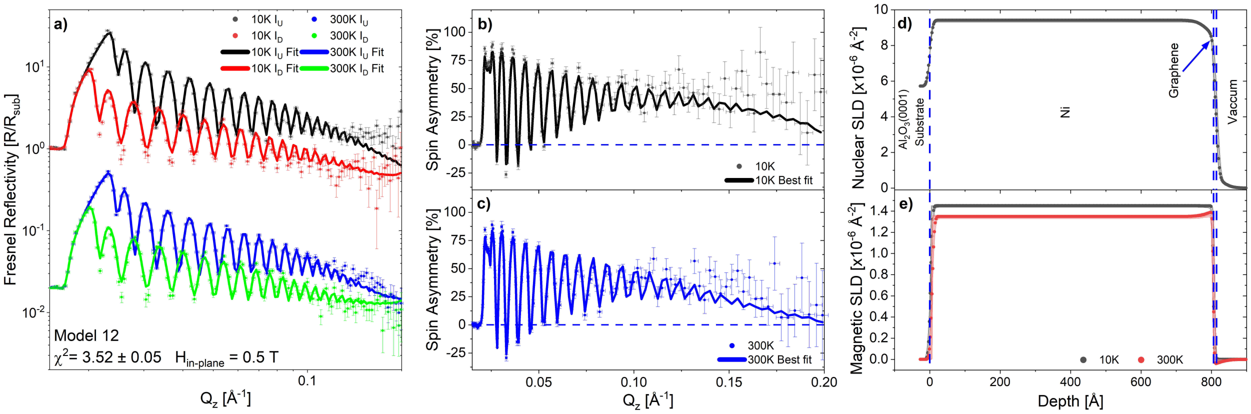

FIG. S15. PNR Model 12: a less realistic version of Model 10 for completeness. The Ni layer has a dead layer at the substrate interface only. The Ni has been split into two continuous Ni layers to allow a gradient across the layer. The graphene layer is constrained so it cannot be thinner than 2 monolayers of graphene in line with the SEM and Raman results. The graphene is set to be non-magnetic. (a) Fresnel reflectivity for 10 K and 300 K, (b) and (c) show the spin asymmetries, (d) is the nuclear scattering length density (nSLD) profile and (e) the magnetic scattering length density (mSLD) profile. The grey banded regions around the SLD lines are the 95% Bayesian confidence intervals. The room temperature Fresnel reflectivity has been shifted by a factor of 50 for ease of display.

Making the graphene roughness conformal with the Ni roughness is the basis for Model 14, shown in Figure S17. This model is identical to Model 8 in all other ways except for this detail. This produces a fit within the error of the χ^2 of model 8, but slightly worse. However, the top surface of the sample appears more physical and removes the sharp drop due to the Ni roughness being greater than the graphene roughness and thickness. Interestingly this further constraint keeps the graphene layer thickness at 2 monolayers of graphene (approx 7.3 Å) without having to fix its priors. Therefore, there is no need to restrain this in line with the SEM and Raman data to two monolayers as was done for Model 9.

Model 14 also has the further effect that it causes the graphene magnetic moment to split such that at 300 K the $m_{\mathrm{Gr300\,K}}$ = 0.03 [0.00, 0.16] μ_B/atom and at 10 K it is $m_{\mathrm{Gr10\,K}}$ = 0.41 [0.16, 0.52] μ_B/atom. This is significantly different to Model 9 where the moments are effectively the same at both temperatures $m_{\mathrm{Gr10\,K}}$ = 0.53 [0.52, 0.54] μ_B/atom and $m_{\mathrm{Gr300\,K}}$ = 0.53 [0.51, 0.54] μ_B/atom, respectively. It should be noted that the XMCD result is the upper estimate for the total graphene magnetic moment at 300 K $m_{\mathrm{XMCD\,Gr300\,K}}$ = 0.47 μ_B/atom, so does not rule the PNR result out.

A final observation on the rotated-domain graphene/Ni data set is that it is noticeable how the data has an upturn at the highest Q in the 10 K data set that is clearly missing in the 300 K data set, marked in Figure S17 by red and blue circles. For this to happen, there would need to be a reflective layer on the surface of the sample present only at low temperature. The

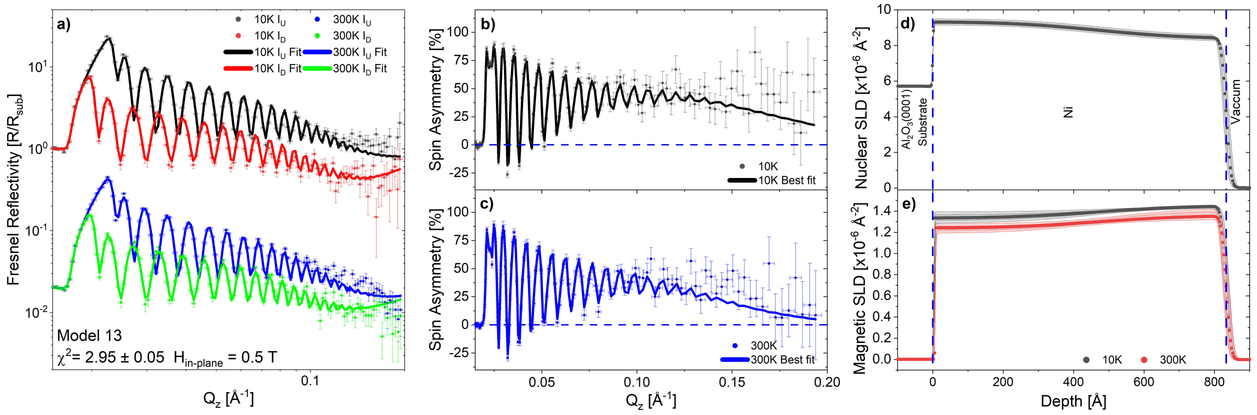

FIG. S16. PNR Model 13: a less realistic version of Model 11. The Ni layer has a dead layer at the substrate interface only. The Ni has been split into two continuous Ni layers to allow a gradient across the layer, and the graphene layer has been removed. (a) Fresnel reflectivity for 10 K and 300 K, (b) and (c) show the spin asymmetries, (d) is the nuclear scattering length density (nSLD) profile and (e) the magnetic scattering length density (mSLD) profile. The grey banded regions around the SLD lines are the 95% Bayesian confidence intervals. The room temperature Fresnel reflectivity has been shifted by a factor of 50 for ease of display.

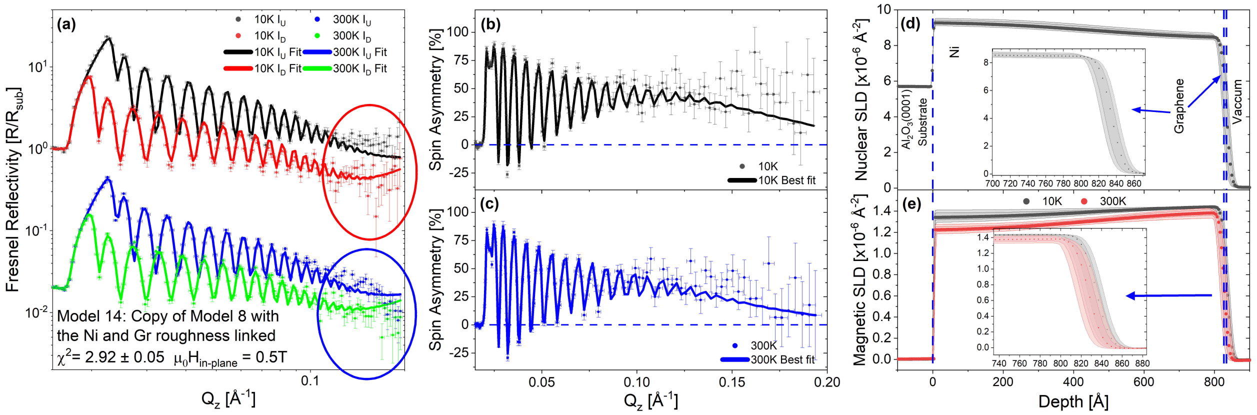

FIG. S17. PNR Model 14: A revised version of Model 8. In this case the roughness of the graphene layer has been set to match the roughness of the top Ni layer directly below, making the two interfaces fully conformal (a) Fresnel reflectivity for 10 K and 300 K, (b) and (c) show the spin asymmetries, (d) is the nuclear scattering length density (nSLD) profile and (e) the magnetic scattering length density (mSLD) profiles. The grey banded regions around the SLD lines are the 95% Bayesian confidence intervals. The room temperature Fresnel reflectivity has been shifted by a factor of 50 for ease of display.

cryostat used to collect this data is an Oxford Instruments helium flow cryostat with 30 mbar of Helium exchange gas. This requires multiple pump and purge procedures when mounting the sample to ensure there is no trapped air or water in the sample volume. However, if this procedure is rushed or there is a small leak, it is possible to condense liquid air or other gaseous condensates onto the surface of the sample. This has been observed in this cryostat on previous experiments in the extreme case of having not removed any air before cooling to 5 K, hence the rationale for taking this into account.

This case was modeled as a liquid air layer with a starting density of 0.87 g/cm^3 with a ratio of elements similar to air (i.e. 78% N, 21% O and 1% Ar).[37] However, we can't say with any certainty what this layer is composed of, so the density value was allowed to vary by ±100%. This condensate layer was only utilized in the model for the 10 K data and was not present in the 300 K part of the model. All other structural parameters were shared as in model 9, where the result are shown in Figure S18.

This dramatically affects the χ^2 value, improving it considerably over Model 9. The 10 K fit has a clear upturn due to the inclusion of a small ≈ 23 Å condensate layer shown in panels (a) and (d) of Figure S18. The density of this layer drops by a third to 0.63 g/cm^3, however, since we don't know what this layer truly consists of, we cannot learn much from this other than there is a thin low density layer at 10 K. Therefore, model 15 was very similar to model 9 except for the addition of the condensate layer at 10 K, which still presents the issue of the top interface roughnesses not being conformal. On the other hand, model 16 displayed in Figure S19 has the addi-

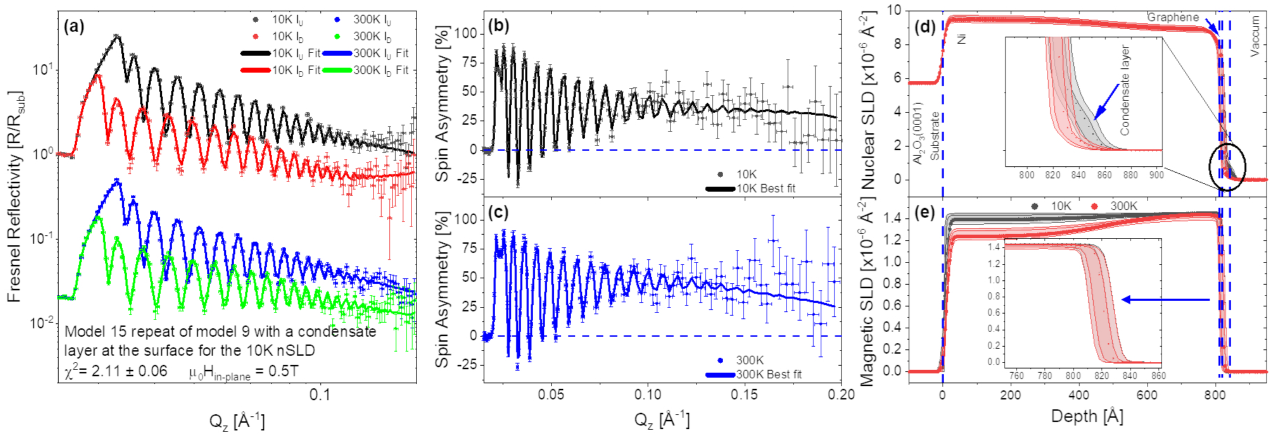

FIG. S18. PNR Model 15: A revised version of Model 9 where a liquid air "condensate" layer has been added to the surface of the 10 K model only, not included in the 300 K model. All other parameters are the same as in model 9 (a) Fresnel reflectivity for 10 K and 300 K, (b) and (c) show the spin asymmetries, (d) is the nuclear scattering length density (nSLD) profiles for 10 K and 300 K. The inset is a zoomed-in region of the top interface showing the condensate layer at 10 K. (e) The magnetic scattering length density (mSLD) profiles. The grey banded regions around the SLD lines are the 95% Bayesian confidence intervals. The room temperature Fresnel reflectivity has been shifted by a factor of 50 for ease of display.

tional constraint of the top Ni layer and the graphene roughnesses being conformal as in model 14. This produces a χ^2 value slightly worse than model 15 but has a similar effect to model 14 on the magnetic moments, splitting them apart at 300 K and 10 K, as compared to them being almost identical in model 15. The main results for the best models 9, 14, 15 and 16 are summarized in Table S3.

We were faced with the conundrum of having 2 models with similar χ^2 values. Taking into account the additional evidence provided by the SEM, Raman, XMCD and some basic physical conditions for roughnesses allows us to qualitatively select the model with two monolayers of graphene that are magnetic and has conformal roughness with the Ni layer below it, i.e. model 16.

Ideally, we would like a more rigorous quantitative measure applicable to the PNR fits to show that this is the most likely case, rather than just stating the assumptions. It is possible to obtain a more quantitative measure by refitting the models using a nested sampler[39,40] to obtain the Bayesian evidence term. Nested sampling takes up vastly more computing time than the MCMC methods used to initially fit the data, hence it is prohibitive to do the majority of the fitting using this method. Therefore it was only used to compare models with very similar χ^2's and it is hard to make a clear judgement between them as to validity. Here we used the UltraNest package.[38]

The details of how nested sampling works are beyond the scope of this work. We direct the interested reader to the work of Skilling *et al*,[39] where nested sampling was developed as a method of Bayesian evidence determination. Primarily we used the work of McCluskey *et al*,[41] who deploys nested sampling for analysing neutron reflectivity data for soft matter experiments. They obtained the Bayesian evidence term and used it to compare different complexities in the models and which most meaningfully fit the data.

This approach avoids the risk of over-fitting as the Bayesian evidence is derived from an integral in parameter space and therefore scales with the number of parameters. It is akin to having a built-in Occam's razor which means that any additional free parameters added to the model must significantly improve the likelihood of the fit in order for the evidence term to show an improvement. However, it is important also to note that the accuracy of the determined evidence depends on the prior probabilities chosen for each of the free parameters and therefore care should be taken to ensure that these are meaningful.[41] All shared parameters between the models have strictly the same priors (uniform ranges and distribution). It is the setting of new additional parameter priors, in our case the graphene thickness and magnetism, as maximum and minimum ranges (assuming a uniform probability distribution as in our case) via our secondary evidence (SEM, Raman and XMCD) that allows us to get some measure of how including this information in the fit gives some validity to the models even when they have very similar χ^2 values. Hence, the Bayesian evidence allows for the comparison of two models, with different numbers of parameters, given that the data they are applied to is the same.

The evidence term is outputted as a negative log-likelihood.[39] In this case, the larger the number is, the more probable the model is. For example, -2 is more probable than -5. There are ways of further utilising the evidence term, but we refer the reader to the literature with regards to these.[42–44]

Models 9-16 were refitted in this manner following the outline by McCluskey *et al*.[41] The evidence terms derived from these fits are shown in Figure S20 (c) and summarized in Table S2. Figure S20 (a) displays the normalized χ^2 values as a function

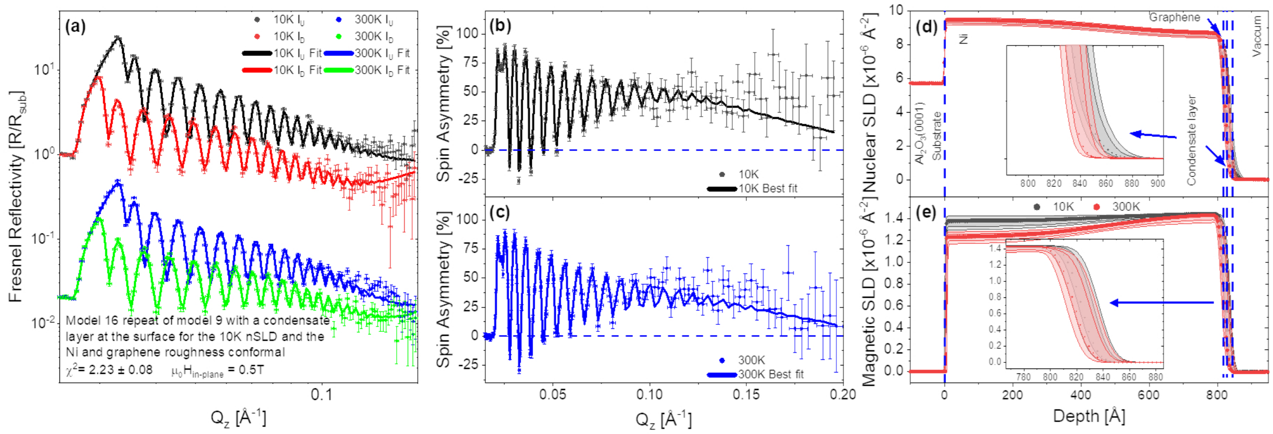

FIG. S19. PNR Model 16: A revised version of Model 9 where a liquid air "condensate" layer has been added to the surface of the 10 K model only, not included in the 300 K model. All other parameters are the same as in model 9 with the addition that the top Ni layer and Graphene roughnesses have been linked to make them conformal (a) Fresnel reflectivity for 10 K and 300 K, (b) and (c) show the spin asymmetries, (d) is the nuclear scattering length density (nSLD) profiles for 10 K and 300 K and (e) the magnetic scattering length density (mSLD) profiles. The grey banded regions around the SLD lines are the 95% Bayesian confidence intervals. The room temperature Fresnel reflectivity has been shifted by a factor of 50 for ease of display.

TABLE S2. Comparison of model parameters, χ^2 values and Bayesian evidence terms.

Model Number	Number of fitted Parameters	χ^2	χ^2 error	Logz (Evidence)	Logz$_{err}$
9	28	2.73	± 0.06	-868.0	± 0.3
10	30	2.86	± 0.06	-915.0	± 0.7
11	27	2.79	± 0.05	-1097.6	± 0.5
12	26	3.52	± 0.05	-888.7	± 0.4
13	23	2.95	± 0.05	-927.1	± 0.4
14	27	2.92	± 0.05	-917.4	± 0.9
15	31	2.11	± 0.06	-698.2	± 0.4
16	32	2.23	± 0.06	-723.8	± 0.7

Table of Evidence: The logz(Evidence) value that is least negative is the most probable model

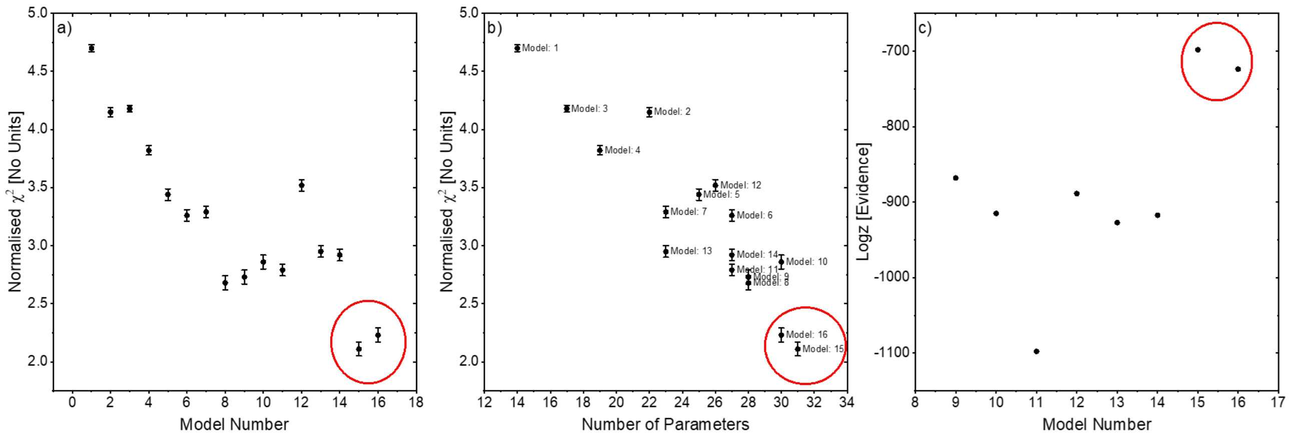

FIG. S20. Panels (a) and (b) show the trends in χ^2 vs the model number and number of parameters, respectively. Models 15 and 16 have the lowest (best) χ^2 values. However, Model 15 is dismissed as the roughness of the graphene was allowed to be non conformal to the Ni as previously reasoned to be non-physical. (c) Bayesian evidence term as computed by the UltraNest nested sampler package.[38] The least negative values are the ones that are most probable, in this case Models 15 and 16. The error bars in panel (c) are smaller than the data points.

of the model number and number of the fitted parameters. It is abundantly clear from this that Model 15 and 16, with the

TABLE S3. Comparison of models 9, 14, 15 and 16. The values in the parenthesis are the lower and upper 95% Bayesian confidence limits.[27]

		FM Layer1 + FM Layer2		Graphene	
Model	Temperature [K]	Thickness [nm]	Magnetic moment [μ_B/atom]	Thickness [nm]	Magnetic moment [μ_B/atom]
Model 9	10 K	80.6(80.2, 81.3)	0.60(0.60, 0.61)	0.86(0.81, 1.04)	0.53(0.52, 0.54)
	300 K		0.57(0.56, 0.57)		0.53(0.51, 0.54)
Model 14	10 K	82.7(81.4, 83.7)	0.62(0.61, 0.62)	0.78(0.3, 1.1)	0.48(0.16, 0.52)
	300 K		0.58(0.57, 0.58)		0.00(0.00, 0.16)
Model 15	10 K	81.2(80.2, 81.9)	0.60(0.60, 0.61)	0.82(0.81, 1.16)	0.53(0.52, 0.54)
	300 K		0.57(0.57, 0.58)		0.51(0.47, 0.53)
Model 16	10 K	82.8(80.8, 82.8)	0.61(0.61, 0.62)	0.82(0.81, 1.2)	0.41(0.28, 0.48)
	300 K		0.58(0.57, 0.58)		0.23(0.02, 0.41)

10 K condensate layer, are more probable than the previous models even with the addition of the extra layers parameters. The results also show that model 15 and 16 are very close, but model 15 is slightly better than 16 probabilistically.

At this point we need to consider models 15 and 16 in light of the initial questions restated below:

Can the model confirm that there is a graphene layer present and primarily is it magnetic? Can the magnitude of the magnetic moment be measured with any certainty?

Both models confirm the presence of a graphene layer and that it is magnetic, in agreement with the other characterisation techniques. Where we struggle is with the magnitude of the moment. The use of the physical constraint of setting the graphene layer roughness equal to that of the Ni layer below it greatly effects the values of the moments, splitting them and making the 95% confidence interval much wider. However, while this approach is arguably more physical (please see the reasoning for model 14 above), the nested sampler clearly shows it is less probably than allowing the roughnesses free rein to vary. The XMCD taken at 300 K indicates an upper limit of the total moment in graphene to be $\approx 0.5 \pm 0.1$ μ_B/atom assuming a 20% accuracy. This is bigger than the 300 K value of 0.23 [0.02,0.41] μ_B/atom from model 16 PNR fit. However, it should be noted that the XMCD value is the upper bound of the moment. Hence the actual value can be lower than this, which lends weight to model 16 for the PNR. So to overrule the nested sampler result, we need a very solid reason. We reasoned for Model 14 that we cannot envision a set of circumstances where the graphene layer would not follow the Ni layer roughness profile below it if that Ni roughness is greater in size than the Gr layer thickness. The graphene is never more than $\approx$ 8 Å thick for all 4 models (9, 14, 15 and 16), sitting on a reliable Ni roughness of $\approx$ 20 Å if the Ni and graphene roughnesses are not linked or $\approx$ 10 Å if they are. Hence the extra freedom of model 15 seems to be driving things non-physical. Considering the fact that the XMCD is an upper bound and does not rule out a smaller moment at 300 K, model 16 appears the most physical or to violate the fewest physical conditions. But we accept this last point is right on the limit and possibly beyond the statistics of the data.

In summary, the decision on the best PNR model was taken by considering the information obtained from the complementary techniques (SEM, Raman spectroscopy and XMCD). Therefore, model 16 was used to estimate the magnetic moment induced in graphene in the rotated-domain graphene/Ni sample. The same model was then used to fit the PNR data of the other systems; epitaxial graphene/Ni and graphene/Ni_9Mo_1 samples. The only exceptions were that the condensate layer was removed from the epitaxial graphene fit as the data contained no evidence for it. The Ni_9Mo_1 data fit also minimised the 10 K condensate layer upon fitting such that a better fit was achieved by removing it. It should be noted that the upturn in the Ni_9Mo_1 data is present at both 300 K and 10 K and is suspected to be a neutron background effect and not a condensate layer as in the rotated graphene.

(1) Shlimak, I.; Haran, A.; Zion, E.; Havdala, T.; Kaganovskii, Y.; Butenko, A. V.; Wolfson, L.; Richter, V.; Naveh, D.; Sharoni, A.; Kogan, E.; Kaveh, M. Raman Scattering and Electrical Resistance of Highly Disordered Graphene. *Physical Review B* **2015**, *91*, 045414.

(2) Allard, A.; Wirtz, L. Graphene on Metallic Substrates: Suppression of The Kohn Anomalies in The Phonon Dispersion. *Nano Letters* **2010**, *10*, 4335–4340.

(3) Malard, L. M.; Pimenta, M. A.; Dresselhaus, G.; Dresselhaus, M. S. Raman Spectroscopy in Graphene. *Physics Reports* **2009**, *473*, 51–87.

(4) Ferrari, A. C.; Basko, D. M. Raman Spectroscopy as a Versatile Tool for Studying The Properties of Graphene. *Nature Nanotechnology* **2013**, *8*, 235–246.

(5) Cabrero-Vilatela, A.; Weatherup, R. S.; Braeuninger-Weimer, P.; Caneva, S.; Hofmann, S. Towards a General Growth Model for Graphene CVD on Transition Metal Catalysts. *Nanoscale* **2016**, *8*, 2149–2158.

(6) Ferrari, A. C. Raman Spectroscopy of Graphene and Graphite: Disorder, Electron-phonon Coupling, Doping and Nonadiabatic Effects. *Solid State Communications* **2007**, *143*, 47–57.

(7) Reichardt, S.; Wirtz, L. *Optical Properties of Graphene*; WORLD SCIENTIFIC, 2017; pp 85–132.

(8) Ferrari, A. C.; Meyer, J. C.; Scardaci, V.; Casiraghi, C.; Lazzeri, M.; Mauri, F.; Piscanec, S.; Jiang, D.; Novoselov, K. S.; Roth, S.; Geim, A. K. Raman Spectrum of Graphene and Graphene Layers. *Physical Review Letters* **2006**, *97*, 187401.

(9) O'Brien, W. L.; Tonner, B. P. Orbital and Spin Sum Rules in X-ray Magnetic Circular Dichroism. *Physical Review B* **1994**, *50*, 12672–12681.

(10) Wang, H.; Bryant, C.; Randall, D. W.; LaCroix, L. B.; Solomon, E. I.; LeGros, M.; Cramer, S. P. X-ray Magnetic Circular Dichroism Sum Rule Analysis of the Blue Copper Site in Plastocyanin. A Probe of Orbital and Spin Angular Momentum. *The Journal of Physical Chemistry B* **1998**, *102*, 8347–8349.

(11) Chen, C. T.; Idzerda, Y. U.; Lin, H.-J.; Smith, N. V.; Meigs, G.; Chaban, E.; Ho, G. H.; Pellegrin, E.; Sette, F. Experimental Confirmation of The X-ray Magnetic Circular Dichroism Sum Rules for Iron and Cobalt. *Physical Review Letters* **1995**, *75*, 152–155.

(12) Idzerda, Y. U.; Chen, C. T.; Lin, H. J.; Tjeng, H.; Meigs, G. Application

of Magnetic Circular Dichroism to Magnetic Thin-films. *Physics B* **1995**, *208-209*, 746–750.

[13] Amemiya, K.; Yokoyama, T.; Yonamoto, Y.; Matsumura, D.; Ohta, T. O K-edge X-ray Magnetic Circular Dichroism of Atomic O Adsorbed on an Ultrathin Co/Cu(100) Film: Comparison with Molecular CO on Co/Cu(100). *Physical Review B* **2001**, *64*, 132405.

[14] Sun, Z.; Zhan, Y.; Shi, S.; Fahlman, M. Energy level Alignment and Interactive Spin Polarization at Organic/Ferromagnetic Metal Interfaces for Organic Spintronics. *Organic Electronics: physics, materials, applications* **2014**, *15*, 1951–1957.

[15] Wende, H.; Scherz, A.; Sorg, C.; Baberschke, K.; Gross, E. K. U.; Appel, H.; Burke, K.; Minár, J.; Ebert, H.; Ankudinov, A. L.; Rehr, J. J. XMCD Analysis Beyond Standard Procedures. *AIP Conference Proceedings* **2007**, *882*, 78–82.

[16] Vogel, J.; Fontaine, A.; Cros, V.; Petroff, F. Structure and Magnetism of Pd in Pd/Fe Multilayers Studied by X-ray Magnetic Circular Dichroism at The Pd L2,3sedges. *Physical Review B - Condensed Matter and Materials Physics* **1997**, *55*, 3663–3669.

[17] Vita, H.; Böttcher, S.; Leicht, P.; Horn, K.; Shick, A. B.; Máca, F. Electronic Structure and Magnetic Properties of Cobalt Intercalated in Graphene on Ir(111). *Physical Review B - Condensed Matter and Materials Physics* **2014**, *90*, 1–10.

[18] Huang, D. J.; Jeng, H. T.; Chang, C. F.; Guo, G. Y.; Chen, J.; Wu, W. P.; Chung, S. C.; Shyu, S. G.; Wu, C. C.; Lin, H. J.; Chen, C. T. Orbital Magnetic Moments of Oxygen and Chromium in CrO2. *Physical Review B* **2002**, *66*, 174440.

[19] Pelzl, J.; Meckenstock, R.; Spoddig, D.; Schreiber, F.; Pflaum, J.; Frait, Z. Spin-orbit-coupling Effects on g-value and Damping Factor of The Ferromagnetic Resonance in Co and Fe Films. *Journal of Physics Condensed Matter* **2003**, *15*.

[20] Gibaud, A.; Vignaud, G.; Sinha, S. K. The correction of geometrical factors in the analysis of X-ray reflectivity. *Acta Crystallographica Section A* **1993**, *49*, 642–648.

[21] Gibaud, A.; Vignaud, G.; Sinha, S. K. The correction of geometrical factors in the analysis of X-ray reflectivity. *Acta Crystallographica Section A* **1993**, *49*, 642–648.

[22] Maranville, B.; Ratcliff II, W.; Kienzle, P. Reductus. `https://www.nist.gov/ncnr/data-reduction-analysis/reflectometry-software`, 2018; `https://www.nist.gov/ncnr/data-reduction-analysis/reflectometry-software`.

[23] Maranville, B.; Ratcliff II, W.; Kienzle, P. *reductus*: a stateless Python data reduction service with a browser front end. *Journal of Applied Crystallography* **2018**, *51*, 1500–1506.

[24] ORSO: Open Reflectometry Standards Organisation. `https://www.reflectometry.org/`, `https://www.reflectometry.org/`.

[25] Caruana, A. Fitting lab XRR data to use with NR. `https://www.youtube.com/watch?v=nNSPWyuhFQ0`, 2020; `https://www.youtube.com/watch?v=nNSPWyuhFQ0`.

[26] Kienzle, P.; Maranville, B.; O'Donovan, K.; Ankner, J.; Berk, N.; Majkrzak, C. `https://www.nist.gov/ncnr/reflectometry-software`, 2017; `https://www.nist.gov/ncnr/reflectometry-software`, Accessed: (28-09-2021).

[27] Kienzle, P.; Krycka, J.; Patel, N.; Sahin, I. `https://bumps.readthedocs.io/en/latest/`, 2011; `https://bumps.readthedocs.io/en/latest/`, Accessed: (28-09-2021).

[28] Abelés, F. La théorie générale des couches minces. *J. Phys. Radium* **1950**, *11*, 307 – 309.

[29] Névot, L.; Croce, P. Caractérisation Des Surfaces Par Réflexion Rasante de Rayons X. Application A L'etude Du Polissage de Quelques Verres Silicates. *Revue de Physique Appliquée* **1980**, *15*, 761 – 779.

[30] Koutsioubas, A. Model-independent Recovery of Interfacial Structure from Multi-contrast Neutron Reflectivity Data. *Journal of Applied Crystallography* **2019**, *52*, 538–547.

[31] Kienzle, P.; Krycka, J.; Patel, N.; Sahin, I. `https://refl1d.readthedocs.io/en/latest/guide/fitting.html#reporting-results`, 2017; `https://refl1d.readthedocs.io/en/latest/guide/fitting.html#reporting-results`, Accessed:(28-09-2021).

[32] McCluskey, A. R.; Caruana, A. J.; Kinane, C. J.; Armstrong, A. J.; Arnold, T.; Cooper, J. F. K.; Cortie, D. L.; Hughes, A. V.; Moulin, J.-F.; Nelson, A. R. J.; Potrzebowski, W.; Straostin, V. Advice on describing Bayesian analysis of neutron and X-ray reflectometry. 2022; `https://arxiv.org/abs/2207.10406`.

[33] NIST Centre for Neutron Research Scattering Length Density Calculator. `https://www.ncnr.nist.gov/resources/sldcalc.html`, `https://www.ncnr.nist.gov/resources/sldcalc.html`.

[34] Density of Graphene. `https://covalentmetrology.com/wp-content/uploads/2021/10/XQTIA002EN-A_Graphene_Characterization.pdf`.

[35] Kittel, C. *Introduction to Solid State Physics*, 8th ed.; Wiley, 2004.

[36] `https://www.webelements.com/compounds/aluminium/dialuminium_trioxide_alpha.html`.

[37] `https://en.wikipedia.org/wiki/Liquid_air`.

[38] Buchner, J. UltraNest - A Robust, General Purpose Bayesian Inference Engine. *The Journal of Open Source Software* **2021**, *6*, 3001.

[39] Skilling, J. Nested Sampling. *AIP Conference Proceedings* **2004**, *735*, 395–405.

[40] Buchner, J. Nested Sampling Methods. 2021.

[41] McCluskey, A. R.; Cooper, J. F. K.; Arnold, T.; Snow, T. A General Approach to Maximise Information Density in Neutron Reflectometry Analysis. *Machine Learning: Science and Technology* **2020**, *1*, 035002.

[42] Kass, R. E.; Raftery, A. E. Bayes Factors. *Journal of the American Statistical Association* **1995**, *90*, 773–795.

[43] Makowski, D.; Ben-Shachar, M. S.; Chen, S. H. A.; Lüdecke, D. Indices of Effect Existence and Significance in The Bayesian Framework. *Frontiers in Psychology* **2019**, *10*.

[44] Trotta, R. Bayes in the sky: Bayesian inference and model selection in cosmology. *Contemporary Physics* **2008**, *49*, 71–104.
